# Debunking highly prevalent health misinformation using audio dramas delivered by WhatsApp: evidence from a randomised controlled trial in Sierra Leone

**DOI:** 10.1136/bmjgh-2021-006954

**Published:** 2021-11-09

**Authors:** Maike Winters, Ben Oppenheim, Paul Sengeh, Mohammad B Jalloh, Nance Webber, Samuel Abu Pratt, Bailah Leigh, Helle Molsted-Alvesson, Zangin Zeebari, Carl Johan Sundberg, Mohamed F Jalloh, Helena Nordenstedt

**Affiliations:** 1 Department of Global Public Health, Karolinska Institutet, Stockholm, Sweden; 2 Center on International Cooperation, New York University, New York, New York, USA; 3 Metabiota, San Francisco, California, USA; 4 FOCUS1000, Freetown, Sierra Leone; 5 College of Medicine and Allied Health Sciences, Freetown, Sierra Leone; 6 Department of Economics, Finance and Statistics, Jönköping International Business School, Jönköping, Sweden; 7 Department of Physiology and Pharmacology, Karolinska Institutet, Stockholm, Sweden

**Keywords:** typhoid and paratyphoid fevers, malaria, public health, epidemiology, randomised controlled trial

## Abstract

**Introduction:**

Infectious disease misinformation is widespread and poses challenges to disease control. There is limited evidence on how to effectively counter health misinformation in a community setting, particularly in low-income regions, and unsettled scientific debate about whether misinformation should be directly discussed and debunked, or implicitly countered by providing scientifically correct information.

**Methods:**

The Contagious Misinformation Trial developed and tested interventions designed to counter highly prevalent infectious disease misinformation in Sierra Leone, namely the beliefs that (1) mosquitoes cause typhoid and (2) typhoid co-occurs with malaria. The information intervention for group A (n=246) explicitly discussed misinformation and explained why it was incorrect and then provided the scientifically correct information. The intervention for group B (n=245) only focused on providing correct information, without directly discussing related misinformation. Both interventions were delivered via audio dramas on WhatsApp that incorporated local cultural understandings of typhoid. Participants were randomised 1:1:1 to the intervention groups or the control group (n=245), who received two episodes about breast feeding.

**Results:**

At baseline 51% believed that typhoid is caused by mosquitoes and 59% believed that typhoid and malaria always co-occur. The endline survey was completed by 91% of participants. Results from the intention-to-treat, per-protocol and as-treated analyses show that both interventions substantially reduced belief in misinformation compared with the control group. Estimates from these analyses, as well as an exploratory dose–response analysis, suggest that direct debunking may be more effective at countering misinformation. Both interventions improved people’s knowledge and self-reported behaviour around typhoid risk reduction, and yielded self-reported increases in an important preventive method, drinking treated water.

**Conclusion:**

These results from a field experiment in a community setting show that highly prevalent health misinformation can be countered, and that direct, detailed debunking may be most effective.

**Trial registration number:**

NCT04112680.

**Key questions**

**What is already known?**

- Health-related misinformation is highly prevalent and highly damaging.
- Randomised trials to counter real-world misinformation remain rare, with most evidence to date being limited to high-income settings.

**What are the new findings?**

- Two narrative audio dramas were tested via WhatsApp in Freetown, Sierra Leone; the first explicitly mentioned and debunked typhoid-related misinformation, the second focused only on providing scientifically correct information.
- Both interventions effectively reduced belief in misinformation as well as improved knowledge and self-reported protective behaviours, but stronger effects were achieved by explicitly citing and debunking misinformation.

**What do the new findings imply?**

- Explicitly addressing why misinformation is wrong via narrative public health messaging may prove effective in countering infodemics.

## Introduction

Misinformation can be as contagious as a virus—sometimes more. And like a virus, misinformation can be fatal. There is strong evidence that misinformation can reduce protective actions, encourage risky behaviours and promote the spread of infectious disease.^1 2^ The WHO has described the current COVID-19 pandemic as an ‘infodemic’, pointing to the overabundance of (mis)information.^3 4^ Widespread misinformation has posed significant challenges to the control of the pandemic, introducing (and amplifying) uncertainty about the importance and efficacy of non-pharmaceutical interventions such as masking and social distancing, as well as safety and efficacy of vaccines for SARS-CoV-2.^2 3 5^ The public health challenges posed by misinformation go far beyond COVID-19. Vaccine hesitancy, driven by online misinformation, has played a role in the recurrence of preventable diseases, notably measles.^6–8^

The rapid rise in the use of social media has increased the volume and velocity of misinformation, giving the especially virulent narratives a wider reach.^9 10^ Despite the urgent need for tools to counter health-related misinformation, there is limited evidence on which strategies are efficacious. Meta-analyses studying different strategies for countering misinformation found that detailed counterarguments could be effective, especially when they are delivered by a trusted source and in line with recipients’ worldviews and social norms.^11 12^ However, this approach does not always yield reductions in belief in misinformation.^11 12^ This might be explained by the continued influence effect, whereby despite credible alternatives, people still rely on the initial misinformation,^13–17^ or via a number of cognitive biases through which repeated exposure to information can strengthen its cognitive availability or appeal, raising the risk that corrective messaging inadvertently strengthens belief in misinformation.^18–20^ Fortunately, evidence thus far shows that these types of unwanted side effects of debunking do not always occur.^21 22^ However, many studies have methodological limitations and few use a pre-post randomised controlled design.^21^ An alternative approach to debunking misinformation emphasises providing correct information rather than directly countering misinformation, to avoid spreading the narrative further to people who would otherwise not have come in contact with it and thus increasing their familiarity with the misinformation.^23–27^

To date, there have been very few experimental studies of interventions to reduce misinformation in non-laboratory settings.^13 15 28^ Most studies aiming to test debunking strategies against health and non-health-related misinformation have been carried out using survey experiments, or in laboratory experiments on university campuses, with relatively small sample sizes and subjects including young, mostly female college students.^11 29^ Furthermore, many studies have not been anchored in a real-world context, as the effectiveness of debunking strategies was evaluated by experimentally introducing a piece of misinformation and subsequently countering its content.^23 30^ In summary, there is limited evidence to date to counter already existing misinformation that is prevalent among the public. In addition, as most studies have been carried out in high-income settings, little is known about debunking strategies in low-income settings that are especially vulnerable to infectious disease outbreaks. Studies that have been performed in low-income settings have mainly looked at various forms of health education to increase knowledge and uptake of protective behaviours, as opposed to specifically testing debunking strategies to target health misinformation.^31–34^

In Sierra Leone, there is widespread misinformation regarding typhoid, and in particular, widespread belief that typhoid and malaria are closely related.^35^ Interestingly, people commonly conceptualise typhoid and malaria as a single disease, ‘typhoid-malaria’. The belief structure linking these diseases is complex and varied. Some narratives indicate that malaria weakens the immune system, which in turn leads to typhoid infection; another narrative suggests that ‘typhoid and malaria walk on the same road’ or ‘are friends’, which implies that the diseases have some causal relationship. Finally, some conceptualise typhoid-malaria as a more severe case of malaria, requiring distinct treatment approaches. The notion that typhoid and malaria occur in conjunction is the common denominator across all explanations. The perceived similarity of the two diseases also makes many people believe that typhoid is caused by mosquitoes.

Although typhoid and malaria share symptoms (eg, fever), they are very different diseases: typhoid is caused by bacterial infection, usually transmitted through contaminated food, water and the faecal-oral route. The incidence of typhoid in Sierra Leone is estimated to be low (around 15 000 cases in 2019).^36^ Malaria is a disease spread by parasite-infected mosquitoes and is much more common than typhoid in Sierra Leone, with more than 3.7 million cases estimated in 2019.^36^

Typhoid can be diagnosed through blood culture. However, in Sierra Leone only one hospital currently has the necessary equipment, and resource constraints limit the availability of blood culture for clinical diagnosis.^37^ Instead, the Widal test is commonly used to diagnose typhoid. The Widal test reportedly has low sensitivity, specificity and positive predictive value for typhoid diagnosis,^38 39^ and may cross-react with malaria antigens, raising the risk of a false-positive result for patients with malaria infections.^40^ Confirmed coinfection of malaria and typhoid is rarely observed.^41–43^ However, in Sierra Leone patients are frequently diagnosed in health centres with ‘typhoid-malaria’, often without using a diagnostic test,^44^ which in addition to antimalarials often is treated with antibiotics.^45^ While there are limited data on typhoid diagnosis and related antibiotic usage in Sierra Leone,^44^ the overdiagnosis of typhoid has likely contributed to the unnecessary use of antibiotics, as well as ensuing antibiotic resistance.^46 47^ Countering typhoid misinformation could therefore inform and empower citizens to question a typhoid-malaria diagnosis and potentially avoid unnecessary usage of antibiotics.

## Methods

The Contagious Misinformation Trial (CMT) was a prospective, three-arm, superiority randomised controlled trial that took place within the community in Freetown, the capital of Sierra Leone, in 2019. The CMT investigated the efficacy of two debunking strategies to counter misinformation about typhoid in Freetown, Sierra Leone, by incorporating scientific and risk communication information into four-episode audio dramas (see table 1) delivered via WhatsApp, a widely used instant messaging platform.

**Table 1:** Core messages of audio dramas by intervention group

Episode	Group A: Plausible Alternative	Group B: Avoiding Misinformation
1. Disease	People think there is a disease called typhoid-malaria, but these are two different diseases.	You can get typhoid by itself, without having other diseases.
2. Cause	Typhoid is not caused by mosquitoes, but by contaminated water and food.	Typhoid is caused by contaminated water and food.
3. Prevention	Sleeping under a bednet helps prevent malaria but does not help prevent typhoid. Good hygiene, drinking treated water and cooking food properly help prevent typhoid.	Prevent yourself from getting typhoid by cooking your food properly and drinking only treated water.
4. Repetition	Repetition of core messages of episodes 1–3.	Repetition of core messages in episodes 1–3.

The audio dramas targeting intervention group A (the Plausible Alternative group) explicitly mentioned the misinformation and provided a detailed counterargument. The audio dramas applied to intervention group B (the Avoiding Misinformation group) did not directly discuss the misinformation, and instead only focused on providing scientifically correct information. The control group received audio messages on breast feeding, unrelated to typhoid-malaria. We tested the efficacy of the two interventions using a randomised controlled trial of 736 participants that took place in the community. Comparing the two interventions allows us to examine whether explicitly invoking and discussing misinformation yields superior results in terms of reducing belief in misinformation. We tested two main outcomes:

- The belief that typhoid is caused by mosquitoes.
- The belief that typhoid can only co-occur with malaria.

The study was designed to detect a relative reduction of 15% in belief in misinformation between one of the intervention groups and the control group. Based on pilot testing, we assumed a 50% prevalence of belief in misinformation. A sample size of 170 per group was required to provide power of 0.80 for a one-sided Wald test. Because of the clustered sampling strategy, the intracluster correlation (ICC) can potentially reduce the effective sample size compared with the calculated sample size. Based on a previous study, we assumed an ICC of 0.01^48^ and a design effect of 1.2.^49^ The sample size was expanded to 250 per group in order to address ICC and potential attrition. The postattrition sample size of 668 gives a statistical power of approximately 0.97.^50^

### Recruitment of participants

We selected 21 of the 64 administrative sections in Freetown as trial sites using weighted random sampling without replacement. As these sections vary widely in size (between roughly 600 and 6000 households), each section had a weighted probability of selection proportionate to its size. The weighted random selection was done by a macro written in Visual Basic for Application for Microsoft Excel. During the recruitment phase, three teams consisting of four enumerators and one supervisor visited one section per day for 7 days (7–13 October 2019). Each enumerator recruited nine new participants in each section. Eligible participants were adults (18 years and older), living in Freetown, fluent in Krio, in possession of a phone with WhatsApp and with no hearing impairments (more details about the recruitment can be found in the online supplemental material).

10.1136/bmjgh-2021-006954.supp1 Supplementary data

Participants received 10.000 leones (about US$1) worth of data credit (around 220 MB) per audio message they received, to ensure that the audio messages could be downloaded. All enumerators and supervisors followed the 3-day training both before the recruitment and baseline survey and before the endline survey was conducted. The aim of the training was for the enumerators to understand the purpose of the study, the recruitment, and to practise the translation of the survey to Krio. The survey was constructed and pilot tested in English, and translated both in written and spoken Krio by a certified translator in Freetown.

The data collection for the endline survey was structured in a similar fashion as the baseline survey, with each team of four enumerators and one supervisor visiting one section per day. Enumerators called participants at least 1 day in advance to make appointments. Five extra data collection days were used to visit participants that could not be reached directly (2–13 December 2019).

In Western Area Urban (the district in which Freetown is situated), an estimated 65% of the population has access to the internet, compared with around 38% elsewhere in the country.^51^ This means that the sample in our study is likely wealthier and more highly educated than the general population in Sierra Leone. To strengthen the external validity and understand whether the intervention would work with another mode of administration, we conducted an ancillary analysis with 60 additional participants who did not have WhatsApp but were in possession of a mobile phone (see online supplemental file 2).

10.1136/bmjgh-2021-006954.supp2 Supplementary data

Data collection teams were instructed not to say words like ‘misinformation’ when recruiting participants. Instead, they would explain that the study would aim to understand people’s knowledge about diseases, as knowledge is power. The Krio name for the study was ‘Info Na Pawa’, or ‘Information is power’. After obtaining written informed consent, the baseline survey was administered in Krio.

### Randomisation and masking

After the recruitment and baseline survey, the participants were randomised 1:1:1 across two intervention groups (A (n=246) and B (n=245)) and one control group (n=245). The random allocation sequence was generated by an Excel Macro (created by ZZ), in which the whole sample was treated as one block.

After randomisation, the study team and the participants were blinded to the allocation of the participants. Enumerators were not aware of participants’ intervention condition during the endline survey. Questions about the audio messages (which would have potentially revealed whether the participant was in the intervention or control group) were asked at the end of the survey, so that enumerators would not be biased. After completion of the endline survey, data were anonymised so that the analysis team was blinded to the allocation of the participants as well.

### Intervention

The two intervention groups in the CMT received audio messages that were based on evidence around countering misinformation. The first intervention (group A) was called ‘Plausible Alternative’, and was informed by research showing that offering a plausible alternative to the misinformation has a higher success rate than simply rejecting the misinformation as false.^11^ The second intervention (group B) was called ‘Avoiding Misinformation’, and was motivated by a less explored debunking method, which is to provide correct information without invoking or mentioning misinformation to limit the risk of further spreading misinformation.^23^ We drew on these theories to produce two sets of audio dramas, with four episodes each.

The audio dramas were produced in Krio with the Freetong Players, a well-known actors group in Sierra Leone. The episodes in the Plausible Alternative drama explicitly cited and discussed misinformation around typhoid and malaria, which was subsequently debunked in the episodes. The episodes in the Avoiding Misinformation drama on the other hand did not mention the misinformation at all and instead focused on the correct information regarding typhoid. The audio dramas incorporated local cultural understandings and language regarding typhoid and malaria. The Freetong Players identified themselves at the start of each episode, and the scientific and risk communication messaging in the dramas was delivered by credible characters: physicians and nurses. By sending out four episodes in each intervention group, we ensured repeated exposure to debunking efforts.^23 24^ Every episode had one core message (see table 1) and lasted between 2 and 5 min. (See the English transcripts in the online supplemental information. To listen to the dramas (in Krio) and access the full dataset see: https://data.mendeley.com/datasets/c758p4dtwz/3 52). Participants in the control group received two episodes promoting breast feeding in Krio, which were approximately 1 min long.

### Outcomes

The two main outcomes (ie, to reduce the belief that (1) typhoid is caused by mosquitoes and (2) can only co-occur with malaria) were captured in the baseline and endline survey with yes/no questions and were analysed with intention-to-treat (ITT) and per-protocol analyses. We conducted a dose–response analysis for each primary outcome. As a robustness check we conducted an as-treated analysis of the two primary outcomes.

The study included several secondary outcomes. First, whether either the intervention unintentionally seeded misinformation among participants who held the correct beliefs as baseline. Second, we tested whether the interventions improved knowledge about preventive methods and self-reported practices for typhoid.

### Statistical analysis

Demographic descriptive statistics were tabulated and differences between the intervention and control groups analysed using χ^2^ tests. We carried out an ITT analysis, excluding the participants who were lost to follow-up. The per-protocol analyses only included participants who reported listening to 100% of the episodes (four episodes for the intervention groups, two episodes for the control group). We also conducted sensitivity analyses to ensure that the per-protocol estimates were not confounded by sample selection (see online supplemental material). For the as-treated analysis, the groups were determined based on the endline survey question ‘*Was the audio about typhoid or about breastfeeding?*’

Crude logistic regression models were specified for the ITT, per-protocol and as-treated analyses. Adjusted logistic regression models incorporated sociodemographic covariates, including sex, education, religion, monthly income and age. As a robustness check, we also estimated Ordinary Least Squares (OLS) regression models with robust SEs for the ITT, per-protocol and as-treated analyses; the results were consistent with the analyses presented in the main text and can be found in the online supplemental information. We applied a Bonferroni adjustment to account for multiple hypothesis tests, setting our alpha at 0.025.

We tested whether the interventions unintentionally seeded misinformation by limiting the analysis to respondents who held scientifically correct beliefs at baseline, and modelling whether the treatments lead to an increase in belief in misinformation at endline. We used logistic regression models, comparing the intervention groups to each other, as well as to the control group.

We conducted an exploratory dose–response analysis by building a treatment index based on the number of episodes each respondent reported listened to; indexing these values allows us to compare the dose–response relationship for the intervention groups (who listened to a maximum of four episodes) to the control group (who listened to a maximum of two episodes). We conducted this analysis for the two primary outcomes using crude and covariate-adjusted logistic regression models.

Knowledge about preventive methods was assessed through an index constructed from an open-ended question on preventive methods that was administered in both the baseline and endline survey (‘*Can you name up to 3 ways how you can prevent yourself from getting typhoid?*’). Correct answers such as drinking treated water were awarded one point, incorrect answers such as taking antimalarials received one minus point (see online supplemental material). The total score per participant varied between −3 and +3. The difference between the intervention groups and the control group in preventive knowledge was analysed through crude and adjusted ordinal logistic regression models.

Lastly, we conducted an exploratory analysis of two behavioural outcomes to estimate whether the intervention increased the use of scientifically grounded approaches to reduce the risk of typhoid infection. In the baseline survey, participants were asked whether they currently take actions to avoid getting infected by typhoid. Those answering yes were asked about the type of actions taken through an open-ended question. Similarly, in the endline survey, participants were asked whether they had taken actions in the last 2 months (ie, the time between the baseline and endline survey) to avoid a typhoid infection. Those answering yes received the open-ended question regarding the specific actions they had taken. Crude and adjusted logistic regression models were fitted for two behavioural outcomes: sleeping under a bednet and drinking treated water (with only the latter a scientifically correct approach to reduce typhoid risk). It should be noted that episode 3 in intervention group A mentioned that while sleeping under a bednet does not prevent a typhoid infection, it does help prevent malaria; we therefore expect intervention group A to be less likely to report sleeping under a bednet to prevent typhoid at endline (though no less likely to use a bednet to avoid malaria infection).

Stata MP V.15 was used for the analysis. The online supplemental tables S10–S15 describe sensitivity analyses. The full study protocol and statistical analysis plan can be accessed at ClinicalTrials.gov and the online supplemental file 2. The study was reported in accordance with Consolidated Standards of Reporting Trials.^53^

## Results

**Figure 1:**
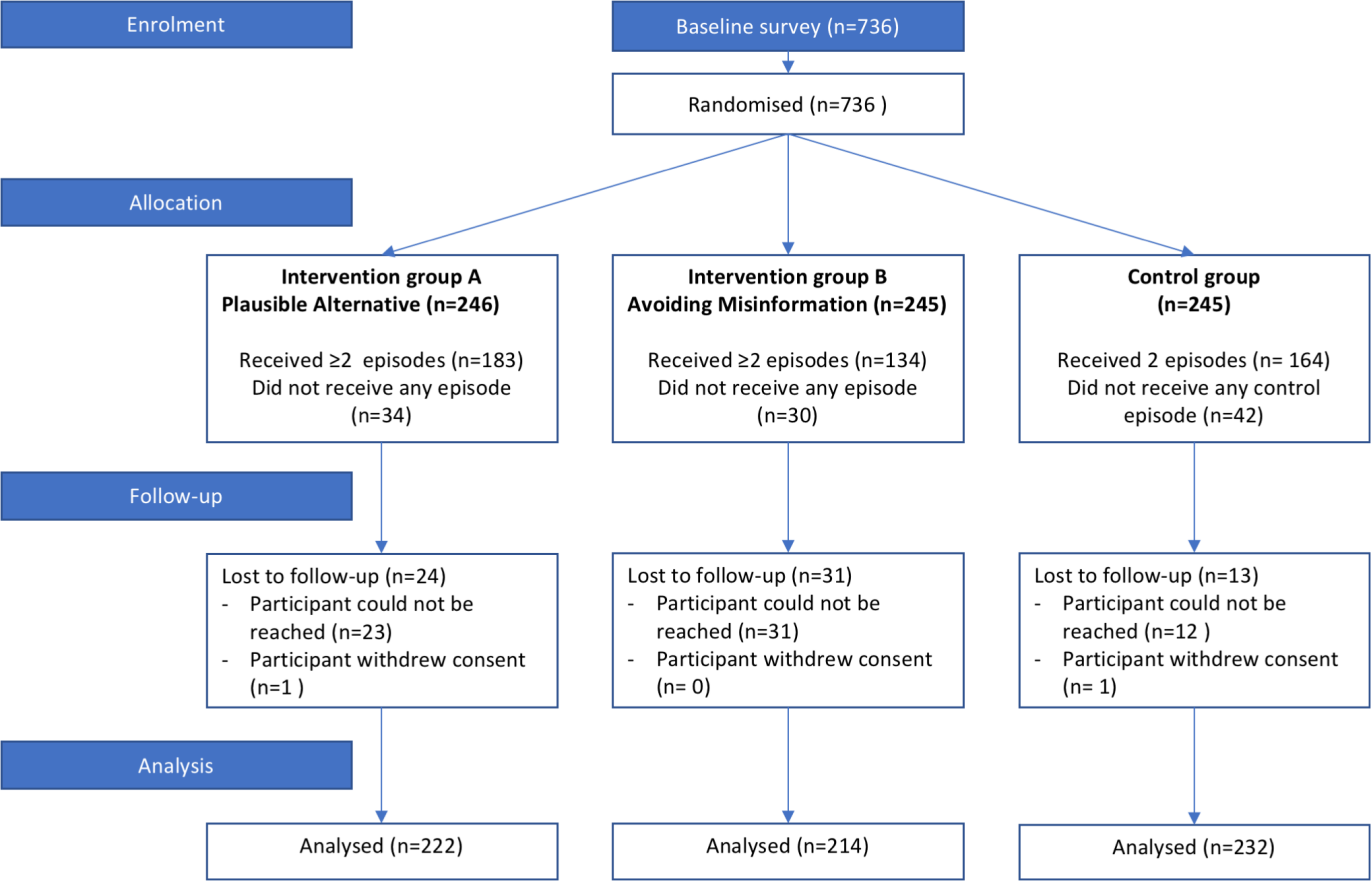
Flow chart of the Contagious Misinformation Trial.

In total, 736 participants in Freetown were enrolled in the CMT. A total of 44 (6%) participants were lost during the intervention period and 24 (3%) participants could not be recontacted for endline data collection, yielding a completion rate of 91% (see figure 1).

Participants who had completed primary education had a slightly higher attrition rate than those who had no formal education, as well as those who had completed secondary and postsecondary education (see online supplemental table S1). The majority of the participants had received secondary education (54%) (see table 2).

**Table 2:** Baseline characteristics of the intervention and control groups

	Group A (n=246)	Group B (n=245)	Control (n=245)	P value*
Age (years)				
18–30	169 (69%)	163 (67%)	151 (62%)	0.147
31–49	59 (24%)	74 (29%)	73 (30%)	
50+	18 (7%)	10 (4%)	21 (9%)	
Sex				
Female	118 (48%)	127 (52%)	130 (53%)	0.499
Male	128 (52%)	118 (48%)	115 (47%)	
Education				
No formal	17 (7%)	9 (4%)	18 (7%)	0.226
Primary	14 (6%)	12 (5%)	12 (5%)	
Secondary	133 (54%)	126 (51%)	142 (58%)	
Postsecondary	82 (33%)	98 (40%)	72 (29%)	
Religion				
Islam	155 (63%)	141 (58%)	149 (61%)	0.461
Christianity	91 (37%)	104 (42%)	96 (39%)	
Income (leones)†				
0–300.000	175 (71%)	158 (64%)	160 (65%)	0.418
300.000–1.000.000	60 (24%)	68 (28%)	69 (28%)	
>1.0000.000	11 (4%)	19 (8%)	16 (7%)	
Typhoid from mosquitoes?				
No	94 (38%)	100 (41%)	93 (38%)	0.648
Yes	123 (50%)	122 (50%)	128 (52%)	
I don’t know	26 (11%)	23 (9%)	23 (9%)	
No response	3 (1%)	0 (0%)	1 (0%)	
Typhoid without malaria?				
No	145 (59%)	146 (60%)	142 (58%)	0.827
Yes	83 (34%)	86 (35%)	89 (36%)	
I don’t know	17 (7%)	13 (5%)	14 (6%)	
No response	1 (0%)	0 (0%)	0 (0%)	

Data are n (%).

*Based on χ^2^ test.

†At the time of the baseline survey 10.000 leones was worth approximately US$1.

Islam was the most common religion among the participants (60%). Two-thirds of the participants earned up to US$30 (300.000 leones) per month. Almost two-thirds of the participants (66%) indicated that they had had typhoid at some point in life. In total, 94% of study participants reported in the baseline survey that they had heard of typhoid-malaria. Belief in misinformation was highly prevalent: at baseline, 51% believed that typhoid is caused by mosquitoes, and 59% believed that typhoid and malaria co-occur (see table 2, online supplemental table S2). There was no statistical difference between the three randomised groups on the demographic variables. At baseline, there was also no statistical difference in belief in misinformation between the intervention and control groups.

### ITT analysis

The belief that typhoid is caused by mosquitoes was significantly reduced in intervention group A compared with the control group in the ITT analysis (group A: adjusted OR (AOR) 0.29, 95% CI 0.18 to 0.47, see table 3 and online supplemental figure S1). In intervention group B, the reduction was not significant (AOR 0.61, 95% CI 0.39 to 0.95, p=0.029).

**Table 3:** Primary outcomes for intervention group A and group B versus control group

	Crude OR (95% CI)	P value	Adjusted* OR (95% CI)	P value
**Typhoid comes from mosquitoes**				
Intention-to-treat (n=583)†				
Group A	0.31 (0.20 to 0.48)	0.000	0.29 (0.18 to 0.47)	0.000
Group B	0.50 (0.33 to 0.77)	0.002	0.61 (0.39 to 0.95)	0.029
Control	Reference	–	Reference	–
Per-protocol (n=210)				
Group A	0.07 (0.02 to 0.19)	0.000	0.06 (0.02 to 0.20)	0.000
Group B	0.31 (0.14 to 0.70)	0.005	0.35 (0.15 to 0.84)	0.019
Control	Reference	–	Reference	–
As-treated (n=419)				
Group A	0.15 (0.08 to 0.27)	0.000	0.13 (0.07 to 0.25)	0.000
Group B	0.33 (0.19 to 0.57)	0.000	0.38 (0.21 to 0.68)	0.001
Control	Reference	–	Reference	–
**Typhoid and malaria co-occur**				
Intention-to-treat (n=618)†				
Group A	0.32 (0.21 to 0.48)	0.000	0.29 (0.19 to 0.45)	0.000
Group B	0.49 (0.33 to 0.73)	0.000	0.55 (0.36 to 0.83)	0.004
Control	Reference	–	Reference	–
Per-protocol (n=220)				
Group A	0.07 (0.03 to 0.18)	0.000	0.06 (0.02 to 0.15)	0.000
Group B	0.16 (0.07 to 0.36)	0.000	0.15 (0.06 to 0.36)	0.000
Control	Reference	–	Reference	–
As-treated (n=434)				
Group A	0.13 (0.08 to 0.23)	0.000	0.12 (0.07 to 0.21)	0.000
Group B	0.25 (0.15 to 0.42)	0.000	0.27 (0.16 to 0.47)	0.000
Control	Reference	–	Reference	–

*Adjusted for sex, education, religion, income and age.

†Complete case analysis. Participants who responded ‘Don’t Know’ or ‘No Response’ in either baseline or endline were excluded. We analysed the impact of intervention assignment on endline non-response (see online supplemental table S9).

The Plausible Alternative intervention (group A) yielded a larger reduction than the Avoiding Misinformation intervention (group B) (AOR 0.46, 95% CI 0.28 to 0.76), though this result does not reach significance in the crude model (table 4). The belief that typhoid co-occurs with malaria was significantly reduced in both intervention groups in the ITT analysis (group A: AOR 0.29, 95% CI 0.19 to 0.45; group B: AOR 0.55, 95% CI 0.36 to 0.83) (table 3 and online supplemental figure S2). Group A showed a greater reduction than group B in the adjusted model (AOR 0.51, 95% CI 0.33 to 0.81, see table 4), but was not significant in the crude model (AOR 0.65, 95% CI 0.43 to 0.98). As a robustness check, we ran the ITT analysis using OLS regression, which yielded similar results (see online supplemental table S3).

**Table 4:** Primary outcomes for intervention group A versus group B

	Crude OR (95% CI)	P value	Adjusted* OR (95% CI)	P value
**Typhoid comes from mosquitoes**				
Intention-to-treat (n=385)†				
Group A	0.61 (0.39 to 0.96)	0.039	0.46 (0.28 to 0.76)	0.002
Group B	Reference	–	Reference	–
Per-protocol (n=130)				
Group A	0.23 (0.08 to 0.73)	0.012	0.15 (0.04 to 0.58)	0.006
Group B	Reference	–	Reference	–
As-treated (n=306)				
Group A	0.47 (0.27 to 0.82)	0.008	0.33 (0.19 to 0.64)	0.001
Group B	Reference	–	Reference	–
**Typhoid and malaria co-occur**				
Intention-to-treat (n=404)†				
Group A	0.65 (0.43 to 0.98)	0.040	0.51 (0.33 to 0.81)	0.004
Group B	Reference	–	Reference	–
Per-protocol (n=133)				
Group A	0.43 (0.15 to 1.25)	0.121	0.32 (0.09 to 1.09)	0.069
Group B	Reference	–	Reference	–
As-treated (n=311)				
Group A	0.55 (0.32 to 0.96)	0.033	0.47 (0.26 to 0.84)	0.011
Group B	Reference	–	Reference	–

*Adjusted for sex, education, religion, income and age.

†Complete case analysis. Participants who responded ‘Don’t Know’ or ‘No Response’ in either baseline or endline were excluded.

### Per-protocol analysis

Similarly, both intervention groups had reduced levels of belief in misinformation under the per-protocol analyses (see tables 3 and 4, online supplemental figures S1 and S2 and online supplemental table S3 for OLS models). The belief that typhoid is caused by mosquitoes was lower in both intervention groups compared with the control group (group A: AOR 0.06, 95% CI 0.02 to 0.20; group B: AOR 0.35, 95% CI 0.15 to 0.84); group A showed sharper declines in the odds than group B (AOR 0.15, 95% CI 0.04 to 0.58). Similarly, the belief that typhoid and malaria co-occur was reduced at endline in the intervention groups compared with the control group (group A: AOR 0.06, 95% CI 0.02 to 0.15; group B: AOR 0.15, 95% CI 0.06 to 0.36). There was no statistical difference between group A and group B (AOR 0.32, 95% CI 0.09 to 1.09).

### As-treated analysis

For the as-treated analysis, 30 participants (13%) were reclassified from control to intervention group A, and 10 participants (3 (1%) in group A and 7 (3%) in group B) from intervention to control. Results are robust to different reclassification techniques (see online supplemental table S4). The as-treated analysis confirmed that participants in both intervention groups were significantly less likely to believe in misinformation. The belief that typhoid is caused by mosquitoes had significantly lower odds in both groups compared with the control group (group A: AOR 0.13, 95% CI 0.07 to 0.25; group B: AOR 0.38, 95% CI 0.21 to 0.68). The belief that typhoid and malaria co-occur had even stronger associations (group A: AOR 0.12, 95% CI 0.07 to 0.21; group B: AOR 0.27, 95% CI 0.16 to 0.47) (see table 3 and online supplemental table S3 for OLS models).

### Seeding misinformation

It is possible that an informational intervention to mitigate misinformation can instead have the undesired effect of ‘seeding’ it, for example, by exposing people who previously held scientifically correct beliefs to factually incorrect beliefs, which then take hold. We analysed whether the intervention unintentionally seeded misinformation among the participants who held the correct beliefs at baseline. Participants in group A were less likely to believe the misinformation at endline compared with the control group, for both the belief that mosquitoes cause typhoid (AOR 0.35, 95% CI 0.15 to 0.81) and the belief that typhoid and malaria co-occur (AOR 0.39, 95% CI 0.18 to 0.82) (see online supplemental table S5). There was no significant difference between group B and the control group for the mosquito belief (AOR 0.70, 95% CI 0.33 to 1.51) and the belief that typhoid and malaria co-occur (AOR 0.58, 95% CI 0.29 to 1.16). There was no difference between group A and group B on both outcomes (mosquito outcome: AOR 0.41, 95% CI 0.15 to 1.05; malaria co-occurrence outcome: AOR 0.72, 95% CI 0.33 to 1.58).

### Dose–response analysis

The dose–response analysis suggests that the group A ‘Plausible Alternative’ intervention was more effective. Both intervention groups significantly reduced their beliefs in the misinformation after having listened to at least two episodes, compared with the control group (see online supplemental table S6). Limiting the analysis to only the two intervention groups, we found a significant interaction between intervention group and dose for the belief that typhoid is caused by mosquitoes (AOR 0.62, 95% CI 0.43 to 0.89), showing that with increased number of episodes, group A performed better than group B (online supplemental table S7). This effect was not observed for the belief that typhoid and malaria co-occur (AOR 0.80, 95% CI 0.58 to 1.11). Furthermore, three episodes of the drama in group A were significantly better at reducing the typhoid-mosquito belief than the four episodes in the group B drama (online supplemental table S8).

### Knowledge about preventive methods

We scored participants’ knowledge about preventive methods on a scale ranging from −3 to +3. The data suggest that both interventions improved study participants’ knowledge: at endline, 67% of the participants in group A scored 1 or higher versus 66% in group B and 51% in the control group. Ordinal logistic regression showed that the two intervention groups scored significantly higher than the control group (group A: AOR 2.19, 95% CI 1.57 to 3.06; group B: AOR 1.79, 95% CI 1.27 to 2.50), but there was no statistically distinguishable effect between the two intervention groups (online supplemental table S9).

### Behavioural outcomes

Exploratory analyses around behavioural outcomes showed that participants in group A were significantly less likely than the control group to report that they were sleeping under a bednet to prevent typhoid infection (AOR 0.43, 95% CI 0.24 to 0.78). There was no statistically significant association for group B (AOR 0.64, 95% CI 0.36 to 1.12) (online supplemental table S9). Both intervention groups had significantly higher odds to report that they were drinking treated water to prevent typhoid infection (group A: AOR 2.78, 95% CI 1.67 to 4.64; group B: AOR 1.77, 95% CI 1.08 to 2.91). There were no statistical differences between intervention groups for either behavioural outcome.

## Discussion

Effectively correcting prevalent public health misinformation is an urgent challenge. The CMT tested two ways of countering prevalent misinformation about typhoid using audio dramas delivered via WhatsApp. Results show that both intervention groups reduced belief in two types of misinformation compared with the control: the belief that typhoid is caused by mosquitoes and the belief that typhoid and malaria co-occur.

Apart from changing the participants’ beliefs in prevalent misinformation, both interventions also positively influenced people’s knowledge and yielded increases in an important protective practice (drinking treated water). It should be noted that this measure was self-reported and might have suffered from social desirability bias. Further studies could gather longitudinal observational data on behavioural risk reduction following (mis)information interventions.

While both interventions reduced belief in misinformation relative to the control group, the Plausible Alternative intervention group, which mentioned misinformation before debunking it, generally experienced stronger improvements in misinformation belief reduction than the Avoiding Misinformation intervention (group B). This is consistent with evidence from laboratory-based studies.^54^ While both interventions contained basic elements of storytelling, the debunking strategy of the Plausible Alternative group incorporated the dramatic element of conflict and debate,^55 56^ which might have made the content ‘stick’ better. Both interventions contained the same volume of scientifically correct, educational content, but intervention group A ‘invested’ additional story time in debunking misinformation; it is possible that increasing the length and scientific detail of the Avoiding Misinformation intervention could increase its effectiveness. Further research on these topics is warranted. However, the Plausible Alternative intervention did not yield statistically significant improvements relative to the Avoiding Misinformation intervention in knowledge of prevention measures or behavioural outcomes.

Contrary to other trials with health communication interventions,^13 14^ we found no evidence that the interventions created negative side effects. Despite concerns that specifically mentioning and debunking misinformation might inadvertently spread scientifically incorrect narratives, we found that the Plausible Alternative intervention did not seed misinformation among those who had previously held correct beliefs. This could be because the risk of spreading misinformation is higher when those audiences are new to the misinformation. However, in our study a large majority of participants (94%) had heard of typhoid-malaria, which may have lowered the risk of seeding the misinformation among those who held the correct beliefs.^22 26^

A major strength of this study is the study design. As a randomised field experiment, the CMT contributes to a small but growing body of research that tests strategies to counter misinformation in a community rather than laboratory setting or survey experiment. The intervention was designed to approximate a real-world public health communication effort, and therefore may have stronger external validity than survey experiments and other commonly used tools to assess the efficacy of informational interventions.

Like other social media, WhatsApp, a widely used messaging platform with global reach, is a platform that can enable the spread of misinformation.^57 58^ At the same time, WhatsApp’s wide reach could be used to deliver effective public health communication campaigns at scale,^58^ while avoiding some of the challenges inherent to radio and television as information channels (eg, information must be ‘consumed’ at time of broadcast, rather than when convenient for the receiver). Further studies are warranted to test corrective messages at scale. These studies might explore the potential for spillover effects, in particular the extent to which health information and educational messaging is shared with others, whether on or off the specific technology platform used to disseminate the intervention. In the case of the CMT, study participants were explicitly instructed not to share the audio dramas. However, real-world information interventions could be much more impactful on a population level if recipients were encouraged to share them, and future studies could explore whether specific types of content, delivery or instructions can encourage ‘productive’ spillover effects of health promotion messaging.

This study also had several limitations. First, despite our ability to monitor message reception and follow-up with study participants, 30% of our participants did not receive or listen to any of the audio episodes. If interventions of this type would be implemented on a larger scale and with less intensive oversight, non-adherence could be higher. Further research could explore the effect of additional reminders and ‘nudges’ on listenership. The endline survey was conducted 8 weeks after the baseline. Future studies should assess the long-term ‘stickiness’ of improvements to knowledge and practices via these and other debunking methods. Furthermore, the misinformation we aimed to counter concerned a specific health-related myth that was not subject to politicised debates. Polarised misinformation might be harder to counter, although the evidence on this is inconclusive thus far.^59 60^ Further experimental work is needed to examine whether the CMT intervention elements would yield similar improvements on polarising misinformation. Similarly, while the misinformation in our study was explicitly debunked in the Plausible Alternative group, it would be of interest to study similar corrective efforts when misinformation consists of implied rather than explicit falsehoods, for instance, through the omission of relevant information.^61^

## Conclusion

These limitations notwithstanding, we have shown that it is possible to reduce belief in misinformation rapidly, even where such beliefs are widely held and reinforced via the health system. A communications strategy that gives room to explain why misinformation is wrong and then provides scientifically correct information, is in line with existing worldviews, delivered by credible sources and gets repeated exposure has the potential to yield desired results without unintentionally seeding misinformation. This list of attributes may sound demanding. However, the results of this field experiment provide some grounds for optimism that even as misinformation becomes more prevalent, there are effective tools at hand to counter its impact and its spread.

## Data Availability

Data are available in a public, open access repository.
