# Supplementary material for: Debunking highly prevalent health misinformation using audio dramas delivered by WhatsApp: evidence from a randomised controlled trial in Sierra Leone
Source: BMJ Glob Health. 2021 Nov 9;6(11):e006954. doi: 10.1136/bmjgh-2021-006954 (PMC8578963; doi:10.1136/bmjgh-2021-006954)
Supplement: Supplementary data [file bmjgh-2021-006954supp001.pdf]

Supplementary Material

Debunking highly prevalent health misinformation using audio dramas delivered by WhatsApp: evidence from a randomised controlled trial in Sierra Leone

This file includes:

Supplementary description of Methods:
- Recruitment of Participants
- Intervention
- Procedures
- Sensitivity Analyses
- Non-WhatsApp Trial Arms

Supplementary Tables:
- Table S1: Lost to follow-up: demographics
- Table S2. Frequencies of the primary outcomes by group
- Table S3: Primary outcomes using OLS regression with robust SEs
- Table S4: As-treated analysis robustness check
- Table S5: Seeding misinformation
- Table S6: Dose response analysis all groups
- Table S7: Dose response analysis intervention groups
- Table S8: Dose response post-hoc analysis between Group A and Group B
- Table S9: Knowledge and practices
- Table S10: Robustness check: contact with other participants
- Table S11: Per-protocol analysis robustness check
- Table S12: Don't Know analysis
- Table S13: Lost to follow-up analysis
- Table S14. Non-WhatsApp baseline characteristics
- Table S15: Non-WhatsApp intention-to-treat and per-protocol analysis

Supplementary Figures:
- Figure S1: Intention-to-treat, per-protocol and as-treated analysis for the belief that typhoid comes from mosquitoes, compared to control group
- Figure S2: Intention-to-treat, per-protocol and as-treated analysis for the belief that typhoid and malaria co-occur, compared to control group

Surveys:
- Baseline Survey Contagious Misinformation Trial
- Endline Survey Contagious Misinformation Trial

Transcripts of interventions:
- Transcripts audio dramas intervention group A
- Transcripts audio dramas intervention group B

Registered Study Protocol & Statistical Analysis Plan (ClinicalTrials.gov NCT04112680)

Winters M, *et al. BMJ Global Health* 2021; 6:e006954. doi: 10.1136/bmjgh-2021-006954

Methods

Recruitment of participants

Every day during the recruitment phase, a data collection team started at the approximate middle of the section (which was determined using printed A3 maps of the sections), where the supervisor threw a pen in the air. The first enumerator walked in the direction of the tip of the pen, the second enumerator in a direction 90 degrees from the first enumerator. The third and fourth enumerator walked in 180 degrees and 270 degrees angle, respectively, from the first enumerator. All enumerators used a skip interval of 15 houses before approaching a household to determine if an eligible adult would be present. Real time review of the GPS points during the recruitment phase was done to ascertain that the geographic spread was indicative of a random selection. Data collection started early in the morning so that people could be encountered at home, before heading to work; it resumed in the late afternoon when most people returned from their day jobs.

Enumerators were instructed to keep a gender balance, recruiting 5 women and 4 men on one day and 4 women and 5 men the next day or vice versa.

Despite the relatively large skip interval, participants might have known each other, and thus posed a risk of contamination within the study. Participants were explicitly asked not to share the audio dramas until after the endline survey was conducted. The endline survey contained a question asking whether they talked to people who had also received audio dramas – 25 participants (3.7%) indicated they did. 3 participants (0.5%) reported receiving audio dramas from someone else. While the risk of contamination is negligible, we fit additional statistical models, omitting the potentially contaminated respondents from the analysis, and find no difference in results (see table S10).

Intervention

We took steps to ensure that the storyline of the two interventions were equally engaging and memorable, to ensure that any observed differences did not result from the narrative "packaging" which contained the health information of interest. Because the Plausible Alternative episodes had elements of conflict and discussion regarding two competing explanations for the illness, we introduced an extra storyline to the Avoiding Misinformation narrative, in which the sister of the main character was about to get married and attendance was unsure due to the typhoid infection. The narratives are thus similar but non-identical, and designed to be equally interesting from a dramatic point of view.

Procedures

WhatsApp Broadcast was used to deliver the audio dramas. This feature allows one message to be sent to a maximum of 256 recipients, without recipients seeing who else received that message. Episode 1 was sent on Monday the 21st of October 2019 with an accompanying message making it clear that it came from the Info Na Pawa study, that these messages should not be forwarded, and providing the number for our helpline. Participants with no WhatsApp were called on Monday and Tuesday the same week and listened to the audio dramas over the phone. This procedure was repeated every week for four weeks for the primary intervention groups and the Non-WhatsApp groups (see S14 and S15 for more info on the Non-WhatsApp group). The control group received the breastfeeding episodes in week 1 and week 3. In week 5 a message was sent to all participants to inform them that they had received all episodes and that enumerators would come back in early December to conduct the endline survey (Dec 2-13, 2019).

By checking and counting blue ticks on WhatsApp the day after (a sign that the recipient has received and has seen the message), we could see to which participants we needed to resend the episode. Through this method, we could also determine that around 70% of the participants had received and seen the 4 episodes in the intervention groups. In the control group, 82% of the participants had received and seen the 2 episodes.

Sensitivity analyses

We conducted a less stringent per-protocol analysis as a robustness check, whereby we included participants who have listened to at least 2 episodes in the intervention groups and at least 1 episode in the control group. Results are in line with the strict per-protocol analysis (see table S11).

It is possible that study participants who followed protocol and listened to the audio dramas differ from those who did not (for example, they could be more receptive to newly introduced information, or more willing to change behaviour). This could cause an upward bias in our estimates of treatment effects. We address this issue via additional analyses: average treatment effects on the treated, and the average potential outcome on the treated by inverse-probability-weighted regression-adjustment (IPWRA). These analyses serve as the sensitivity analyses for the unbiasedness of the per-protocol treatment analysis. The results of these analyses are consistent with the main per-protocol analysis reported above, indicating that our per-protocol results are not confounded by sample selection (data available on request.)

Many participants mentioned not eating chicken to avoid typhoid infection. This response most likely related to a typhoid-scare in 2016, when a large amount of expired imported chicken was dumped on landfills in Freetown and reportedly consumed by some of Freetown's poorest residents - resulting in typhoid (1). This response was coded as 0 for the knowledge score, as it correctly identifies a historical risk factor but not the more general preventive behaviour needed to reduce typhoid infection risk.

Lastly, we conducted a number of exploratory sensitivity analyses. We assessed patterns of item non-response and "don't know" responses on dependent variables across the two survey waves, and found no indication that the two interventions increased "don't know" responses (for example by shifting people from belief in misinformation from indicating uncertainty). On the contrary, we found that for the belief that typhoid co-occurs with malaria, group A had a statistically significant reduction in "don't know" responses, compared to the control group, see table S12. We also analysed whether the results were robust to the exclusion of the small number of respondents who had potentially been contaminated by discussing the intervention messages with others, and found that their exclusion did not impact the results we present here, see table S10. Lastly, we analysed whether the exclusion of the respondents who answered "don't know" or "no response" on either the baseline or the endline survey influenced the intention-to-treat analyses. Results were consistent with the primary intention-to-treat analyses, data are available upon request.

Non-WhatsApp Trial Arms

A smaller-scale parallel study which delivered the audio dramas by phone, rather than WhatsApp, achieved similar results, suggesting that the impacts we observe may be achieved through a range of potential information channels.

The Non-WhatsApp participants were similarly randomised to two intervention groups: Non-WhatsApp group A (n=30) and Non-WhatsApp group B (n=30), which were compared to the main control group. For the Non-WhatsApp comparator study, we analysed the two primary outcomes using the intention-to-treat and per-protocol analyses, comparing the intervention groups to the control group of the WhatsApp participants.

Reference:

1. Kanu K. Condemned Chicken! Your Life or Poverty. Cocorioko. 2016.https://cocorioko.net/condemned-chicken-your-life-or-poverty/ (accessed 7 Nov 2019).

Table S1. Lost to follow-up: demographics

	Included No (%)	Lost to follow-up No (%)	P-value*
**Sex**			
Male	346 (52%)	29 (43%)	0.150
Female	322 (48%)	39 (57%)	
**Education**			
No formal	39 (6%)	5 (7%)	0.001
Primary	30 (4%)	8 (12%)	
Secondary	371 (56%)	30 (44%)	
Post-secondary	228 (34%)	24 (35%)	
**Religion**			
Islam	400 (60%)	45 (66%)	0.312
Christianity	268 (40%)	23 (34%)	
**Income (in Leones)**			
0-300.000	448 (67%)	45 (66%)	0.738
300.000-1.000.000	177 (27%)	20 (29%)	
>1.000.000	43 (6%)	3 (4%)	

*Based on Chi2 test
There was a significant difference in educational level between the participants included in this study and the participants that were lost to follow-up, whereby those lost to follow-up were more likely to have lower educational attainment

Table S2. Frequencies of the primary outcomes by group

Can you get typhoid from mosquitoes?

	**Baseline**			**Endline**		
	**Group A No (%)**	**Group B No (%)**	**Control No (%)**	**Group A No (%)**	**Group B No (%)**	**Control No (%)**
Yes	123 (50)	122 (50)	128 (52)	73 (33)	78 (36)	120 (52)
**No**	94 (38)	100 (41)	93 (38)	145 (65)	128 (60)	98 (42)
I don't know	26 (11)	23 (9)	23 (9)	4 (2)	8 (4)	13 (6)
No response	1 (1)	0 (0)	1 (0)	0 (0)	0 (0)	1 (0)

Can you get typhoid without getting malaria?

	**Baseline**			**Endline**		
	**Group A No (%)**	**Group B No (%)**	**Control No (%)**	**Group A No (%)**	**Group B No (%)**	**Control No (%)**
**Yes**	83 (34)	86 (35)	89 (36)	153 (69)	129 (60)	95 (41)
No	145 (59)	146 (60)	142 (58)	65 (29)	83 (39)	131 (56)
I don't know	17 (7)	13 (5)	14 (6)	4 (2)	2 (1)	5 (2)
No response	1 (0)	0 (0)	0 (0)	0 (0)	0 (0)	1 (0)

Winters M, *et al. BMJ Global Health* 2021; 6:e006954. doi: 10.1136/bmjgh-2021-006954

Table S3. Primary outcomes using OLS regression with robust SEs

	Coef (SEs)	P-value	Adjusted* Coef (SEs)	P-value	Adjusted* Coef (SEs)	P-value
**Typhoid comes from mosquitoes**						
Intention-to-treat						
Group A	-0.25 (0.05)	0.000	-0.24 (0.04)	0.000	-0.14 (0.04)	0.002
Group B	-0.15 (0.05)	0.001	-0.10 (0.05)	0.023	Reference	-
Control	Reference	-	Reference	-	-	-
Per-protocol						
Group A	-0.40 (0.06)	0.000	-0.38 (0.07)	0.000	-0.16 (0.06)	0.007
Group B	-0.23 (0.07)	0.001	-0.20 (0.07)	0.004	Reference	-
Control	Reference	-	Reference	-	-	-
As-treated						
Group A	-0.36 (0.05)	0.000	-0.36 (0.05)	0.000	-0.14 (0.04)	0.002
Group B	-0.23 (0.05)	0.000	-0.20 (0.05)	0.000	Reference	-
Control	Reference	-	Reference	-	-	-
**Typhoid and malaria co-occur**						
Intention-to-treat						
Group A	-0.27 (0.05)	0.000	-0.27 (0.05)	0.000	-0.13 (0.05)	0.005
Group B	-0.17 (0.05)	0.000	-0.14 (0.05)	0.003	Reference	-
Control	Reference	-	Reference	-	-	-
Per-protocol						
Group A	-0.46 (0.06)	0.000	-0.47 (0.07)	0.000	-0.10 (0..06)	0.087
Group B	-0.37 (0.07)	0.000	-0.36 (0.07)	0.000	Reference	-
Control	Reference	-	Reference	-	-	-
As-treated						
Group A	-0.42 (0.05)	0.000	-0.42 (0.05)	0.000	-0.13 (0.05)	0.005
Group B	-0.32 (0.05)	0.000	-0.29 (0.05)	0.000	Reference	-
Control	Reference	-	Reference	-	-	-

*Adjusted for: sex, education, religion, income, age
OLS = Ordinary Least Squares, Coef = Coefficient, SE = Standard error

Winters M, *et al. BMJ Global Health* 2021; 6:e006954. doi: 10.1136/bmjgh-2021-006954

Table S4. As-treated analysis robustness check
In this scenario the participants who were assigned to the control group but said they received messages about typhoid (n=30), were added to intervention Group B (Avoiding Misinformation)

	**Crude OR (95% CI)**	**P-value**	**Adjusted* OR (95% CI)**	**P-value**	**Adjusted* OR (95% CI)**	**P-value**
**Typhoid comes from mosquitoes**						
Group A	0.12 (0.06-0.23)	0.000	0.11 (0.06-0.22)	0.000	0.46 (0.28-0.76)	0.002
Group B	0.33 (0.19-0.56)	0.000	0.35 (0.20-0.62)	0.000	Reference	-
Control	Reference	-	Reference	-	-	-
**Typhoid and malaria co-occur**						
Group A	0.11 (0.06-0.19)	0.000	0.09 (0.05-0.18)	0.000	0.51 (0.33-0.81)	0.004
Group B	0.26 (0.16-0.43)	0.000	0.27 (0.16-0.45)	0.000	Reference	-
Control	Reference	-	Reference	-	-	-

*Adjusted for: sex, education, religion, income, age
OR = Odds Ratio, CI = Confidence Interval

Table S5. Seeding misinformation

	Crude OR (95% CI)	P-value	Adjusted* OR (95% CI)	P-value	Adjusted* OR (95% CI)	P-value
**Typhoid comes from mosquitoes**						
Group A	0.35 (0.16-0.77)	0.008	0.35 (0.15-0.81)	0.014	0.41 (0.16-1.05)	0.064
Group B	0.57 (0.29-1.14)	0.111	0.70 (0.33-1.51)	0.369	Reference	-
Control	Reference	-	Reference	-	-	-
**Typhoid and malaria co-occur**						
Group A	0.41 (0.21-0.82)	0.012	0.39 (0.18-0.82)	0.014	0.72 (0.33-1.58)	0.413
Group B	0.57 (0.30-1.10)	0.093	0.58 (0.29-1.16)	0.123	Reference	-
Control	Reference	-	Reference	-	-	-

*Adjusted for: sex, education, religion, income, age

Table S6. Dose response analysis all groups

**Typhoid comes from mosquitoes**	**Crude OR (95% CI)**	**P-value**	**Adjusted* OR (95% CI)**	**P-value**
No of episodes listened to:				
0	Reference	-	Reference	-
1	0.58 (0.25-1.32)	0.195	0.50 (0.21-1.19)	0.116
2	0.30 (0.18-0.52)	0.000	0.31 (0.18-0.55)	0.000
3	0.11 (0.06-0.21)	0.000	0.13 (0.07-0.25)	0.000
4	0.12 (0.07-0.19)	0.000	0.14 (0.08-0.22)	0.000
**Typhoid and malaria co-occur**	**Crude OR (95% CI)**	**P-value**	**Adjusted* OR (95% CI)**	**P-value**
No of episodes listened to:				
0	Reference	-	Reference	-
1	0.73 (0.33-1.63)	0.446	0.65 (0.29-1.48)	0.307
2	0.33 (0.20-0.58)	0.000	0.36 (0.21-0.61)	0.000
3	0.13 (0.07-0.25)	0.000	0.15 (0.08-0.29)	0.000
4	0.15 (0.10-0.24)	0.000	0.17 (0.11-0.27)	0.000

* Adjusted for: sex, education, religion, income, age
OR = Odds Ratio, CI = Confidence Interval

Table S7. Dose response analysis intervention groups

	Crude OR (95% CI)	P-value	Adjusted* OR (95% CI)	P-value
**Typhoid comes from mosquitoes**				
Dose (0-4)	0.57 (0.47-0.69)	0.000	0.65 (0.52-0.82)	0.000
Intervention group				
Group A	1.30 (0.64-2.66)	0.470	1.06 (0.45-2.52)	0.888
Group B	Reference	-	Reference	-
Interaction (Dose x Group AB)	0.71 (0.53-0.96)	0.028	0.62 (0.43-0.89)	0.009
**Typhoid and malaria co-occur**				
Dose (0-4)	0.52 (0.43-0.64)	0.000	0.54 (0.43-0.67)	0.000
Intervention group				
Group A	0.79 (0.39-1.60)	0.510	0.71 (0.32-1.55)	0.386
Group B	Reference	-	Reference	-
Interaction (Dose x Group AB)	0.83 (0.61-1.13)	0.235	0.80 (0.58-1.11)	0.187

*Adjusted for: sex, education, religion, income, age

Table S8. Dose response post-hoc analysis between Group A and Group B
Number of episodes listened to, Odds Ratios with 95% Confidence Intervals

**Typhoid comes from mosquitoes**	**Group B**				
**Group A**	**0**	**1**	**2**	**3**	**4**
**0**	1.06 (0.45-2.52)	1.63 (0.76-3.46)	2.48 (1.22-5.05)*	3.79 (1.81-7.95)*	5.79 (2.51-13.37)*
**1**	0.43 (0.02-0.92)	0.66 (0.35-1.24)	1.00 (0.56-1.80)	1.53 (0.83-2.84)	2.34 (1.13-4.85)*
**2**	0.17 (0.08-0.37)*	0.27 (0.14-0.50)*	0.41 (0.23-0.72)*	0.62 (0.34-1.14)	0.95 (0.46-1.95)
**3**	0.07 (0.03-0.17)*	0.11 (0.05-0.23)*	0.16 (0.08-0.33)*	0.25 (0.12-0.52)*	0.38 (0.17-0.87)*
**4**	0.03 (0.01-0.08)*	0.04 (0.02-0.11)*	0.07 (0.03-0.16)*	0.10 (0.04-0.25)*	0.16 (0.06-0.42)*
**Typhoid and malaria co-occur**	**Group B**				
**Group A**	**0**	**1**	**2**	**3**	**4**
**0**	0.71 (0.32-1.55)	1.32 (0.67-2.62)	2.47 (1.29-4.73)*	4.61 (2.30-9.22)*	8.60 (3.86-19.14)*
**1**	0.30 (0.15-0.61)*	0.57 (0.32-1.01)	1.06 (0.62-1.81)	1.98 (1.10-3.54)*	3.69 (1.83-7.44)*
**2**	0.13 (0.06-0.26)*	0.24 (0.14-0.43)*	0.45 (0.27-0.77)*	0.85 (0.48-1.50)	1.58 (0.80-3.15)
**3**	0.06 (0.03-0.12)*	0.10 (0.05-0.21)*	0.20 (0.10-0.37)*	0.36 (0.19-0.71)*	0.68 (0.32-1.46)
**4**	0.02 (0.01-0.06)*	0.04 (0.02-0.10)*	0.08 (0.04-0.19)*	0.16 (0.07-0.36)*	0.29 (0.12-0.72)*

*p-value<0.025

Table S9. Knowledge and practices

	Crude OR (95% CI)	P-value	Adjusted* OR (95% CI)	P-value	Adjusted* OR (95% CI)	P-value
**Knowledge**						
Group A	2.19 (1.57-3.04)	0.000	2.19 (1.57-3.06)	0.000	1.24 (0.88-1.75)	0.226
Group B	2.11 (1.51-2.94)	0.000	1.79 (1.27-2.50)	0.001	Reference	-
Control	Reference	-	Reference	-	-	-
**Sleeping under a bednet**						
Group A	0.43 (0.24-0.77)	0.004	0.43 (0.24-0.78)	0.005	0.66 (0.35-1.27)	0.213
Group B	0.56 (0.32-0.96)	0.035	0.64 (0.36-1.12)	0.118	Reference	-
Control	Reference	-	Reference	-	-	-
**Drinking treated water**						
Group A	2.74 (1.68-4.48)	0.000	2.78 (1.67-4.64)	0.000	1.57 (0.92-2.68)	0.100
Group B	1.97 (1.23-3.16)	0.005	1.77 (1.08-2.91)	0.023	Reference	-
Control	Reference	-	Reference	-	-	-

*Adjusted for: sex, education, religion, income, age

Table S10. Robustness check: contact with other participants
In this robustness check, we excluded the participants who indicated to have contact with other participants of the Info Na Pawa study (n=25, 3.7%) and those who received the audio dramas from someone else (n= 3, 0.5%).

	Crude OR (95% CI)	P-value	Adjusted* OR (95% CI)	P-value	Adjusted* OR (95% CI)	P-value
**Typhoid comes from mosquitoes**						
**Intention-to-treat**						
Group A	0.29 (0.19-0.46)	0.000	0.28 (0.17-0.46)	0.000	0.47 (0.28-0.79)	0.004
Group B	0.47 (0.30-0.72)	0.001	0.57 (0.36-0.90)	0.015	Reference	-
Control	Reference	-	Reference	-	-	-
**Per-protocol**						
Group A	0.06 (0.02-0.18)	0.000	0.05 (0.02-0.18)	0.000	0.12 (0.03-0.58)	0.008
Group B	0.26 (0.11-0.61)	0.002	0.29 (0.12-0.74)	0.009	Reference	-
Control	Reference	-	Reference	-	-	-
**As-treated**						
Group A	0.14 (0.08-0.26)	0.000	0.12 (0.06-0.24)	0.000	0.47 (0.28-0.79)	0.004
Group B	0.30 (0.17-0.53)	0.000	0.35 (0.19-0.64)	0.001	Reference	-
Control	Reference	-	Reference	-	-	-
**Typhoid and malaria co-occur**						
**Intention-to-treat**						
Group A	0.32 (0.21-0.49)	0.000	0.29 (0.19-0.46)	0.000	0.53 (0.33-0.84)	0.007
Group B	0.48 (0.32-0.71)	0.000	0.54 (0.36-0.82)	0.004	Reference	-
Control	Reference	-	Reference	-	-	-
**Per-protocol**						
Group A	0.08 (0.03-0.20)	0.000	0.06 (0.02-0.17)	0.000	0.36 (0.10-1.26)	0.109
Group B	0.15 (0.07-0.35)	0.000	0.14 (0.06-0.36)	0.000	Reference	-
Control	Reference	-	Reference	-	-	-
**As-treated**						
Group A	0.12 (0.07-0.22)	0.000	0.11 (0.06-0.20)	0.000	0.53 (0.33-0.84)	0.007
Group B	0.23 (0.13-0.39)	0.000	0.25 (0.14-0.45)	0.000	Reference	-
Control	Reference	-	Reference	-	-	-

*Adjusted for: sex, education, religion, income, age
OR = Odds Ratio, CI = Confidence Interval

Winters M, *et al. BMJ Global Health* 2021; 6:e006954. doi: 10.1136/bmjgh-2021-006954

Table S11. Per-protocol analysis robustness check
Scenario 2: considering all participants who have listened to at least 2 episodes in the intervention groups to be per-protocol, or at least 1 episode in the control group

	Crude OR (95% CI)	P-value	Adjusted* OR (95% CI)	P-value	Adjusted* OR (95% CI)	P-value
**Typhoid comes from mosquitoes**						
Group A	0.12 (0.06-0.23)	0.000	0.12 (0.06-0.24)	0.000	0.23 (0.11-0.49)	0.000
Group B	0.38 (0.22-0.66)	0.001	0.50 (0.28-0.88)	0.016	Reference	-
Control	Reference	-	Reference	-	-	-
**Typhoid and malaria co-occur**						
Group A	0.14 (0.07-0.25)	0.000	0.13 (0.07-0.24)	0.000	0.39 (0.20-0.76)	0.006
Group B	0.29 (0.17-0.48)	0.000	0.33 (0.20-0.57)	0.000	Reference	-
Control	Reference	-	Reference	-	-	-

*Adjusted for: sex, education, religion, income, age
OR = Odds Ratio, CI = Confidence Interval

Table S12. Don't know analysis

In this analysis, we tested whether the interventions had any impact on the proportion of respondents who indicated uncertainty regarding the endline measures of interest: whether typhoid comes from mosquitoes, and whether it only co-occurs with malaria. Specifically, we were interested in whether the interventions shifted more respondents from initial uncertainty (e.g. answering "don't know" at baseline) to having a clear viewpoint at endline (e.g. answering either yes or no), and additionally the converse (e.g. whether either intervention shifted people from yes/no at baseline to "don't know" at endline). We generated a variable taking a value of +1 if a respondent shifted from don't know to a certain answer at endline, and -1 if they shifted from certainty to uncertainty, and 0 if no change. A positive statistically significant value shows that participants who said 'Don't Know' at baseline were more likely to have a certain answer (Yes/No) at endline. A negative statistically significant value would indicate that participants who had a certain answer (Yes/No) at baseline were more likely to answer 'Don't Know' at endline. We found that Intervention Group A led to an increase in certain answers from baseline to endline from the question regarding whether typhoid is caused by mosquitos; neither intervention had an impact on the question regarding the co-occurrence of typhoid and malaria.

	**Coefficient (SE*)**	**P-value**
**Typhoid comes from mosquitoes**		
Group A	0.456 (0.059-0.853)	0.025
Group B	0.253 (-0.152-0.657)	0.218
Control	Reference	-
**Typhoid and malaria co-occur**		
Group A	0.119 (-0.361-0.599)	0.621
Group B	0.346 (-0.198-0.891)	0.207
Control	Reference	-

OLS regression
*SE = Standard Error

Table S13. Lost to follow-up analysis
In this analysis, the answers of those lost to follow-up were regarded as unchanged at endline (i.e. they were regarded to be the same as the baseline answers)

**Typhoid comes from mosquitoes**	**Crude OR (95% CI)**	**P-value**	**Adjusted* OR (95% CI)**	**P-value**
Group A	0.34 (0.22-0.52)	0.000	0.32 (0.20-0.51)	0.000
Group B	0.57 (0.37-0.86)	0.008	0.64 (0.41-0.99)	0.047
Control	Reference	-	Reference	-
**Typhoid and malaria co-occur**	**Crude OR (95% CI)**	**P-value**	**Adjusted* OR (95% CI)**	**P-value**
Group A	0.34 (0.23-0-51)	0.000	0.32 (0.21-0.49)	0.000
Group B	0.54 (0.37-0.79)	0.001	0.59 (0.40-0.87)	0.008
Control	Reference	-	Reference	-

* Adjusted for: sex, education, religion, income, age
OR = Odds Ratio, CI = Confidence Interval

Table S14. Non-WhatsApp baseline characteristics

	Group A (n=30)	Group B (n=30)	Control (n=245)	Total	P-value*
**Age**					
18-30 years	17 (57%)	10 (33%)	151 (62%)	178 (58%)	0.031
31-49 years	11 (37%)	14 (47%)	73 (30%)	98 (32%)	
50+ years	2 (7%)	6 (20%)	21 (9%)	29 (10%)	
**Sex**					
Female	17 (57%)	12 (40%)	130 (53%)	159 (52%)	0.365
Male	13 (43%)	18 (60%)	115 (47%)	146 (48%)	
**Education**					
No formal	3 (10%)	3 (10%)	18 (7%)	24 (8%)	0.001
Primary	6 (20%)	8 (27%)	12 (5%)	26 (9%)	
Secondary	18 (60%)	14 (47%)	142 (58%)	174 (57%)	
Post-secondary	3 (10%)	5 (17%)	72 (29%)	80 (26%)	
**Religion**					
Islam	18 (60%)	17 (57%)	149 (61%)	149 (61%)	0.930
Christianity	12 (40%)	13 (43%)	96 (39%)	96 (39%)	
**Income (Leones)**					
0-300.000	20 (67%)	16 (53%)	160 (65%)	196 (64%)	0.519
300.000-1.000.000	9 (30%)	10 (33%)	69 (28%)	88 (29%)	
>1.000.000	1 (3%)	4 (13%)	16 (7%)	21 (7%)	
**Typhoid from mosquitoes?**					
No	9 (30%)	10 (33%)	93 (38%)	112 (37%)	0.916
**Yes**	19 (63%)	18 (60%)	128 (52%)	165 (54%)	
I don't know	2 (7%)	2 (7%)	23 (9%)	27 (9%)	
No response	0 (0%)	0 (0%)	1 (0.4%)	1 (0.3%)	
**Typhoid without malaria?**					
**No**	25 (83%)	20 (67%)	142 (58%)	187 (61%)	0.098
Yes	4 (13%)	9 (30%)	89 (36%)	102 (33%)	
I don't know	1 (3%)	1 (3%)	14 (6%)	16 (5%)	
No response	0 (0%)	0 (0%)	0 (0%)	0 (0%)	

*Fisher's exact test
There was a significant difference between groups for Age (whereby the WhatsApp control group was older than the Non WhatsApp Intervention groups) and Education (the Non WhatsApp intervention groups were less highly educated than the WhatsApp control group)

Table S15. Non-WhatsApp intention-to-treat and per-protocol† analysis

	**Crude OR (95% CI)**	**P-value**	**Adjusted* OR (95% CI)**	**P-value**	**Adjusted* OR (95% CI)**	**P-value**
**Typhoid comes from mosquitoes**						
**Intention-to-treat**						
Group A	0.24 (0.10-0.60)	0.002	0.20 (0.07-0.52)	0.001	1.57 (0.33-7.36)	0.569
Group B	0.19 (0.07-0.52)	0.001	0.21 (0.07-0.64)	0.006	Reference	-
Control	Reference	-	Reference	-	-	-
**Per-protocol**						
Group A	0.23 (0.08-0.64)	0.005	0.16 (0.05-0.49)	0.001	0.77 (0.14-4.32)	0.766
Group B	0.21 (0.07-0.62)	0.005	0.20 (0.06-0.67)	0.009	Reference	-
Control	Reference	-	Reference	-	-	-
**Typhoid and malaria co-occur**						
**Intention-to-treat**						
Group A	0.16 (0.06-0.42)	0.000	0.13 (0.05-0.35)	0.000	0.55 (0.10-2.90)	0.480
Group B	0.25 (0.10-0.62)	0.003	0.26 (0.10-0.70)	0.008	Reference	-
Control	Reference	-	Reference	-	-	-
**Per-protocol**						
Group A	0.13 (0.04-0.42)	0.001	0.08 (0.02-0.28)	0.000	0.26 (0.03-2.07)	0.205
Group B	0.25 (0.09-0.67)	0.006	0.21 (0.07-0.66)	0.007	Reference	-
Control	Reference	-	Reference	-	-	-

*Adjusted for: sex, education, religion, income, age
OR = Odds Ratio, CI = Confidence Interval

†The per-protocol analysis includes respondents from Groups A and B who reported that they listened to at least 2 episodes, and in the Control Group, at least 1 message

Among the Non-WhatsApp participants, belief in misinformation was higher at baseline, with 63% of group A and 60% in group B reporting the belief that typhoid is caused by mosquitoes. In group A 83% and in group B 67%, respectively believed that typhoid and malaria co-occur. The intention-to-treat analyses showed that the belief that typhoid is caused by mosquitoes was reduced in both intervention groups, compared to the control group (group A: AOR 0.20, 95% CI 0.07-0.52, group B: AOR 0.21, 95% CI 0.07-0.64). Similarly, the two intervention groups were less likely to believe that typhoid and malaria co-occur, compared to the control group (group A: AOR 0.13, 95% CI 0.05-0.35, group B: AOR 0.26, 95% CI 0.10-0.70). Per-protocol analyses yielded similar results.

Supplementary Figures

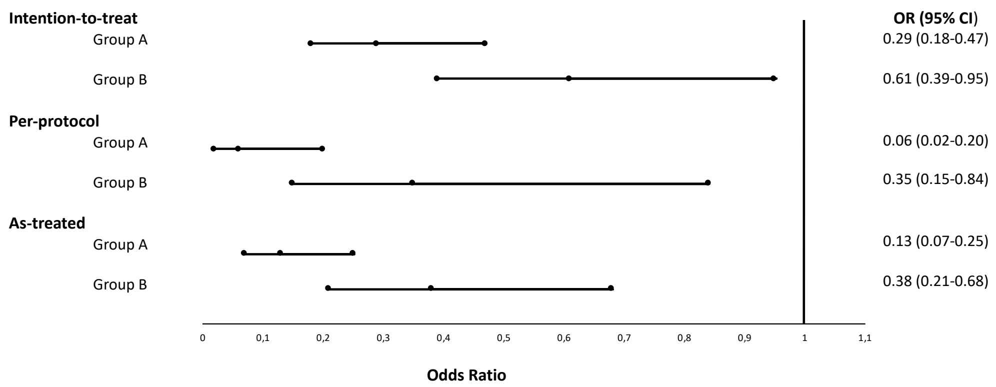

Figure S1. Intention-to-treat, per-protocol and as-treated analysis for the belief that typhoid comes from mosquitoes, compared to control group

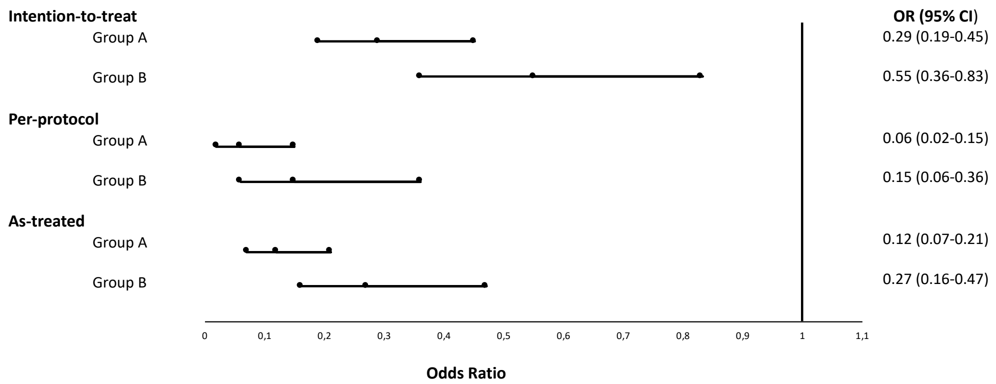

Figure S2. Intention-to-treat, per-protocol and as-treated analysis for the belief that typhoid and malaria co-occur, compared to control group

Winters M, *et al. BMJ Global Health* 2021; 6:e006954. doi: 10.1136/bmjgh-2021-006954

**Baseline Survey Contagious Misinformation Trial**		
**No**	**Question**	**Answer alternatives**
1	Name of enumerator	
2	Does the participant have WhatsApp?	Yes<br>No → end
3	Does the participant want to be part of the study?	Yes<br>No → end
4	Is the study phone number programmed in the participant's phone?	Yes → Q6<br>No → Q5
5	If no, why not?	
6	Did the participant sign the informed consent form?	Yes<br>No → end
7	First name	
8	Last name	
9	Address	
10	Describe house / address / landmark	
11	WhatsApp phone number	
12	Is your WhatsApp number from Orange, Africell, or Qcell?	Orange<br>Africell<br>Q cell<br>Other:<br>I don't know<br>No answer
13	Other phone number participant can be reached on	
14	Gender	Male<br>Female
15	Age in years	
16	What is your highest level of education?	No formal education<br>Primary education<br>Secondary education<br>Post-secondary education<br>I don't know/not sure<br>No response
17	Average monthly household income from paid job in Leones	
18	What is your religion?	Islam<br>Christianity<br>Other:<br>No response
19	How often do you discuss health issues with family or friends?	Daily<br>Weekly<br>Monthly<br>When necessary<br>Never<br>I don't know/not sure<br>No response
20	Do you know of any diseases that can be spread by being in contact with another person? Do not read the alternatives, select all that apply	Malaria<br>HIV<br>Ebola<br>Typhoid<br>Measles<br>Lassa Fever<br>Cholera<br>Zika<br>Tuberculosis<br>Leprosy<br>Scabies<br>Sexually transmitted diseases

		Other:
21	Have you every had typhoid?	Yes → Q22<br>No → Q25<br>I don't know/not sure → Q25
22	How did you know you had typhoid? <u>Do not read the alternatives, select all that apply</u>	Diagnosed in a hospital<br>Diagnosed in a health facility other than a hospital<br>Diagnosed in a private clinic<br>From a pharmacy<br>From a health center<br>From a health-worker<br>From a lab<br>Somebody told me<br>From the symptoms<br>I don't know/not sure<br>No response
23	How many times have you had typhoid?	
24	When was the last time you had typhoid?	In the last 3 months<br>In the last 6 months<br>In the last year<br>Longer ago<br>I can't remember<br>No response
25	<u>For data collectors only:</u> Did the respondent mention 'typhoid-malaria' so far?	Yes<br>No
26	How does a person get typhoid? <u>Do not read the alternatives, select all that apply</u>	Mosquitoes<br>After getting malaria<br>Bacteria<br>Virus<br>Parasite<br>Witchcraft/evildoing/sin<br>God or higher power<br>By eating contaminated food<br>By drinking contaminated water<br>Eating with dirty hands<br>Contact between houseflies and food<br>Contact with vomit or stool<br>Drinking too much beer<br>Eating too many oranges<br>Eating peanuts<br>Eating oily foods<br>Not washing off sweat<br>Other:<br>I don't know/not sure<br>No response
27	Can a person get typhoid from mosquitoes?	Yes<br>No<br>I don't know/not sure<br>No response
28	How would you know that someone is infected with typhoid (signs and symptoms)? <u>Do not read the alternative, select all that apply</u>	Weakness<br>Fever<br>Chills<br>Headache<br>Muscle pain<br>Diarrhea<br>Vomiting<br>Abdominal (stomach) pain<br>Constipation<br>Lack of appetite<br>Sore throat<br>Cough

		Rash<br>Difficulty breathing<br>Confusion<br>Dizziness<br>Yellow eyes/yellow urine<br>Other:<br>I don’t know/not sure<br>No response
29	Can you name up to three ways how you can prevent yourself from getting typhoid? Do not read the alternative, select all that apply (max three!)	Sleep under a bednet<br>Drink treated water<br>Wash hands with soap before eating<br>Wash hands before cooking food<br>Wash hands after leaving the toilet<br>Wash food before eating<br>Cook foods thoroughly<br>Eat foods while hot<br>Take anti-malarials<br>Don’t eat too many oranges<br>Don’t eat oily foods<br>Don’t drink too much beer<br>Keep environment clean<br>Other:<br>I don’t know/not sure<br>No response
30	If you think that you or someone in your family has typhoid, what would you do? Do not read the alternative, select all that apply	Go to a health facility/hospital<br>Go to -a traditional healer/traditional birth attendant<br>Get medication from a pharmacy<br>Get antibiotics from a pharmacy<br>Get anti-malarials from a pharmacy<br>Self-medicate<br>Other:<br>I don’t know/not sure<br>No response
31	Let’s say you keep doing what you already do to avoid typhoid, do you think it is likely or unlikely for you to get typhoid in the next year?	Likely<br>Unlikely<br>I don’t know<br>No response
32	Do you currently take actions to avoid getting typhoid?	Yes<br>No → Q34<br>I don’t know<br>No response
33	What kind of actions do you currently take to avoid getting typhoid? Do not read the alternatives, select all that apply	Sleep under a bednet<br>Drink treated water<br>Wash hands with soap before eating<br>Wash hands before cooking food<br>Wash hands after leaving the toilet<br>Wash food before eating<br>Cook foods thoroughly<br>Eat foods while hot<br>Take anti-malarials<br>Don’t eat too many oranges<br>Don’t eat oily food<br>Don’t drink too much beer<br>Keep environment clean<br>Other:<br>I don’t know/not sure<br>No response
34	Would you take actions in the next year to avoid getting typhoid?	Yes<br>No → Q36<br>I don’t know/not sure<br>No response

35	What kind of actions would you take in the next year to avoid getting typhoid? Do not read the alternatives, select all that apply	Sleep under a bednet<br>Drink treated water<br>Wash hands with soap before eating<br>Wash hands before cooking food<br>Wash hands after leaving the toilet<br>Wash food before eating<br>Cook foods thoroughly<br>Eat foods while hot<br>Take anti-malarials<br>Don't eat too many oranges<br>Don't eat oily food<br>Don't drink too much beer<br>Keep environment clean<br>Other:<br>I don't know/not sure<br>No response
36	How does a person get malaria? Do not read the alternatives, select all that apply	Mosquitoes<br>Bacteria<br>Virus<br>Parasite<br>Witchcraft/evildoing/sin<br>God or higher power<br>By eating contaminated food<br>By drinking contaminated water<br>Eating with dirty hands<br>Contact between houseflies and food<br>Contact with vomit or stool<br>Drinking too much beer<br>Eating too many oranges<br>Eating peanuts<br>Eating oily foods<br>Not washing off sweat<br>Other:<br>I don't know/not sure<br>No response
37	Can you get typhoid without getting malaria?	Yes → Q40<br>No → Q38<br>I don't know<br>No response
38	Why do you think that? Do not read the alternative, select all that apply	Typhoid and malaria go together<br>First you get malaria, then typhoid<br>Typhoid is a bad form of malaria<br>Both are caused by mosquitoes<br>Signs and symptoms are the same<br>Other:
39	Where did you get this information? Do not read the alternatives, select all that apply	Health facility/hospital<br>Radio<br>Television<br>Church/mosque/other religious venues<br>Community meetings<br>Newspapers/text messages<br>Traditional leaders<br>Traditional healers<br>Government/Ministry of Health/Well Bodi Ministry<br>Family/friends<br>Doctors<br>Nurses<br>Other health workers<br>Other community workers<br>Other:<br>I don't know/not sure

		No response
40	Record geo location	

**Endline Survey Contagious Misinformation Trial**		
**No**	**Question**	**Answer alternatives**
1	Name enumerator	
2	Trial ID of participant	
3	First name	
4	Last name	
5	WhatsApp phone number (in case of non WhatsApp participant: phone 1)	
6	Trial ID again of participant	
7	What kind of work do you currently do?	Private business (excluding petty traders)<br>Plumber/carpenter/electrician<br>Petty trader<br>Farmer<br>Teacher/lecturer/instructor<br>Public transportation driver (taxi, buses, podapoda)<br>Okada rider<br>Medical or health professional<br>Other governmental employee (not already listed above)<br>Student<br>Unemployed<br>Other:<br>No response
8	How often do you discuss health issues with family or friends? <u>Do not read the alternatives, select all that apply</u>	Daily<br>Weekly<br>Monthly<br>When necessary<br>Never<br>I don't know/not sure<br>No response
9	Have you had typhoid in the last 2 months?	Yes → Q10<br>No → Q11<br>I don't know/not sure
10	How did you know you had typhoid? <u>Do not read the alternatives, select all that apply</u>	Diagnosed in a hospital<br>Diagnosed in a health facility other than a hospital<br>Diagnosed in a private clinic<br>From a pharmacy<br>From a health center<br>From a non-health worker<br>From a lab<br>Somebody told me<br>From the symptoms<br>I don't know/not sure<br>No response
11	How does a person get typhoid? <u>Do not read the alternatives, select all that apply</u>	Mosquitoes<br>After getting malaria<br>Bacteria<br>Virus<br>Parasite<br>Witchcraft/evildoing/sin<br>God or higher power<br>By eating contaminated food<br>By drinking contaminated water<br>Eating with dirty hands<br>Contact between houseflies and food<br>Contact with vomit or stool<br>Drinking too much beer

		Eating too many oranges<br>Eating peanuts<br>Eating oily foods<br>Bad hygiene<br>Other:<br>I don't know/not sure<br>No response
12	Can a person get typhoid from mosquitoes?	Yes<br>No<br>I don't know/not sure<br>No response
13	How would you know that someone is infected with typhoid (signs and symptoms)? Do not read the alternative, select all that apply	Weakness<br>Fever<br>Chills<br>Headache<br>Muscle pain<br>Diarrhea<br>Vomiting<br>Abdominal (stomach) pain<br>Constipation<br>Lack of appetite<br>Sore throat<br>Cough<br>Rash<br>Difficulty breathing<br>Confusion<br>Dizziness<br>Yellow eyes/yellow urine<br>Other:<br>I don't know/not sure<br>No response
14	Can you name up to three ways how you can prevent yourself from getting typhoid? Do not read the alternative, select all that apply	Sleep under a bednet<br>Drink treated water<br>Wash hands with soap before eating<br>Wash hands before cooking food<br>Wash hands after leaving the toilet<br>Wash food before eating<br>Cook foods thoroughly<br>Eat foods while hot<br>Take anit-malarials<br>Don't eat too many oranges<br>Don't eat oily food<br>Don't drink too much beer<br>Clean environment<br>Other:<br>I don't know/not sure<br>No response
15	If you think that you or someone in your family has typhoid, what would you do? Do not read the alternative, select all that apply	Go to a health facility/hospital<br>Go to a traditional healer/traditional birth attendant<br>Get medication from a pharmacy<br>Get antibiotics from a pharmacy<br>Get anti-malarials from a pharmacy<br>Self-medicate<br>Other:<br>I don't know/not sure<br>No response
16	Do you think it is likely or unlikely for you to get typhoid in the next year?	Likely<br>Unlikely<br>I don't know/not sure<br>No response

Winters M, *et al. BMJ Global Health* 2021; 6:e006954. doi: 10.1136/bmjgh-2021-006954

17	Have you taken actions in the last 2 months to avoid getting typhoid?	Yes<br>No → Q19<br>I don't know/not sure<br>No response
18	In what ways have you taken actions to avoid getting typhoid? <u>Do not read the alternatives, select all that apply</u>	Sleep under a bednet<br>Drink treated water<br>Wash hands with soap before eating<br>Wash hands before cooking food<br>Wash hands after leaving the toilet<br>Wash food before eating<br>Cook foods thoroughly<br>Eat foods while hot<br>Take anti-malarials<br>Don't eat too many oranges<br>Don't eat oily food<br>Don't drink too much beer<br>Clean environment<br>Other:<br>I don't know/not sure<br>No response
19	Are you planning to take actions in the next year to avoid getting typhoid?	Yes<br>No → Q21<br>I don't know/not sure<br>No response
20	What kind of actions would you take in the next year to avoid getting typhoid? <u>Do not read the alternatives, select all that apply</u>	Sleep under a bednet<br>Drink treated water<br>Wash hands with soap before eating<br>Wash hands before cooking food<br>Wash hands after leaving the toilet<br>Wash food before eating<br>Cook foods thoroughly<br>Eat foods while hot<br>Take anti-malarials<br>Don't eat too many oranges<br>Don't eat oily food<br>Don't drink too much beer<br>Clean environment<br>Other:<br>I don't know/not sure<br>No response
21	How does a person get malaria? <u>Do not read the alternatives, select all that apply</u>	Mosquitoes<br>Bacteria<br>Virus<br>Parasite<br>Witchcraft/evildoing/sin<br>God or higher power<br>By eating contaminated food<br>By drinking contaminated water<br>Eating with dirty hands<br>Contact between houseflies and food<br>Contact with vomit or stool<br>Drinking too much beer<br>Eating too many oranges<br>Eating peanuts<br>Eating oily foods<br>Not washing off sweat<br>Other:<br>I don't know/not sure<br>No response
22	Can you get typhoid without getting malaria?	Yes, PROBE: 'So you are saying that you can get typhoid without getting malaria?'

		No, PROBE: 'So you are saying that typhoid and malaria come together?'<br>I don't know<br>No response
23	Where did you get this information? Do not read the alternatives, select all that apply	Health facility/hospital<br>Radio<br>Television<br>Church/mosque/other religious venues<br>Community meetings<br>Newspapers/text messages<br>Traditional leaders<br>Traditional healers<br>Government/Ministry of Health/Well Bodi Ministry<br>Family/friends<br>Doctors<br>Nurses<br>Other health workers<br>Other community workers<br>Other:<br>I don't know/not sure<br>No response
24	How many audio messages did you get? Was it 2 or 4 audio messages?	
25	How many audio messages did you listen to?	
26	Was the audio about typhoid or about breastfeeding?	About typhoid → Q27<br>About breastfeeding → Q30
27	Who was the main character in the drama?	Mariama<br>Abu<br>Sullay<br>Other:
28	Where did Mariama possibly get infected with typhoid?	
29	What happened to the main character in the drama? Is the participant describing it correctly?	Yes<br>No<br>Not sure: describe what the participant is saying
30	Did you like the drama?	Yes<br>No
31	Did you discuss the audio messages with friends and/or family?	Yes<br>No
32	Have you received information about typhoid and malaria from other sources in the last 2 months?	Yes → from where?<br>No<br>Other:
33	Have you talked to anyone who has heard or received the Info Na Pawa messages as well?	Yes<br>No<br>Other:
34	Have you received the audio messages from anybody else as well, not just from Info Na Pawa study?	Yes<br>No<br>Other:
35	Do you feel you now know more, less, or the same about typhoid and malaria?	More<br>Less<br>The same<br>Other:
36	For data collectors only: Did the respondent mention 'typhoid-malaria' so far?	Yes<br>No
37	General remarks (data, top ups etc)	

38	Trial ID participant	
39	WhatsApp phone number again	
40	Record geo location	

Transcripts of Audio dramas

Intervention Group A: Plausible Alternative

Group A – Episode 1

{Music playing}

Introduction

My people, listen to this drama! With the Freetong Players International, FOCUS 1000 and the Karolinska Institute in Sweden we bring to you..

Episode One

Abu: Mariama, are you still having a fever?

Mariama: Yes Sir. I am feeling weak and I also have a stomach ache.

Abu: You've been ill for five days, let's go to the hospital.

Mariama: Ok Abu.

{Music playing}

Abu: Nurse … nurse, good morning.

Male Nurse: Yes Sir, good morning.

Abu: I am here with my wife.

Male Nurse: Ok. Madam good morning.

Mariama: Nurse good morning.

Male Nurse: What is your name and how exactly are you feeling?

Mariama: My name is Mariama.

Male Nurse: Ok.

Mariama: I am feeling weak and I have a stomach ache. I also have a fever.

Male Nurse: {Sighing} Your wife has typhoid-malaria.

Abu: I said that.

Male Nurse: Because the symptoms indicates typhoid-malaria.

Abu: That is why I decided to come to the hospital.

Head Nurse: Nurse, I heard you talking about typhoid-malaria.

Male Nurse: Yes Ma'am.

Head Nurse: There is nothing like typhoid-malaria.

Male Nurse: Ok.

Head Nurse: Typhoid is typhoid and malaria is malaria. Have you conducted a test?

Male Nurse: No Ma’am.

Head Nurse: Well, test her for malaria.

Male Nurse: Ok, we are going to do the test now.

Head Nurse: Test her now for malaria.

Male Nurse: Do not worry, it’s just a small prick.

Mariama: {Exclaim}

Head Nurse: Look at this, the patient does not even have malaria. Everything she complained about: weakness, stomach ache and fever indicates that the patient has typhoid. Therefore, we should treat her for typhoid.

Male Nurse: Ok Ma’am.

Head Nurse: Alright, treat her for typhoid. Thereafter, call all the other nurses. I want to talk to you all.

Male Nurse: Yes Ma’am.

Head Nurse: Ok.

Male Nurse: You are going to be okay. Everything will be alright.

Abu: Mariama, we should go home now.

Mariama: Ok.

Abu: Take it easy.

{Music playing}

Female nurse: Yes sister, we are told that you called us.

Male Nurse: Yes Ma’am, we are here.

Head Nurse: Alright. I want to talk about typhoid-malaria. Everybody is talking about typhoid-malaria, typhoid-malaria, typhoid-malaria.

Male Nurse: Exactly.

Head Nurse: There is nothing like typhoid-malaria! Typhoid is typhoid and malaria is malaria.

Male Nurse: Huh?

Head Nurse: These are two different diseases. They might have similar symptoms but they are two different diseases.

Male Nurse: Wow!

Head Nurse: That is why I called you guys to enlighten you.

Male Nurse: Thank you very much sister. Everything that you explained we never knew and this is the first time we hear this information. However, we have learned from what you explained. Thank you very much.

{Music playing}

Group A – Episode 2

{Music playing}

Introduction

My people, listen to this drama! With the Freetong Players International, FOCUS 1000 and the Karolinska Institute in Sweden, we bring to you..

Episode Two

Abu: Mariama!

Mariama: Yes Abu?

Abu: How are you feeling now?

Mariama: I am feeling better now. When I take my medicine, I am getting better.

Abu: Mariama, what causes typhoid?

Mariama: I am sure it is mosquitoes that are responsible for typhoid.

Abu: Don’t you think ground nut and cooking oil are responsible for typhoid?

Mariama: Hmm Abu, it is mosquitoes that cause typhoid.

Abu: You know what, let’s call Sullay and ask him.

Mariama: Okay, let’s call him.

Abu: Sullay, Sullay.

Sullay: Yes brother?

Abu: Sullay, come!

Sullay: Yes, yes brother.

Abu: What causes typhoid? Is it mosquitoes?

Sullay: Brother, I am not sure; I don't know about that. I am not sure it is mosquitoes but I know a well-known doctor, I will meet him and ask him. He will tell me the truth whether it is mosquitoes.

Mariama: Okay Abu.

Sullay: I will meet him.

{Music playing}

Door knocking

Doctor: Who is it?

Sullay: It is Sullay.

Doctor: Sullay, my friend, come in, come in!

Sullay: Ha doc, doc.

Doctor: Long time Sullay.

Sullay: I am here to see you. I want to ask you a question. I want to ask you a question about typhoid and malaria. I know that typhoid and malaria are two different diseases.

Doctor: Of course.

Sullay: I do understand. However, I want to know what causes typhoid. Is it mosquitoes?

Doctor: No, no, no Sullay. Mosquitoes do not cause typhoid. Typhoid is caused by drinking unclean water, and also uncooked food causes typhoid. Also, hygiene; after using the toilet you must wash your hands before touching anything.

Sullay: Doctor, is that all.

Doctor: That's is all Sullay.

Sullay: Okay doctor. Thank you very much doctor. I will give you a call.

Doctor: Okay Sullay.

Sullay: Okay {Sullay laughing}

{Music playing}

Sullay: Brother, aunty, I am back.

Abu: Yes Sullay?

Sullay: I have seen the doctor.

Abu: Sullay, what did the doctor say?

Sullay: He said typhoid is not caused by mosquitoes. He said typhoid is caused by drinking unclean water and eating uncooked food. Thirdly, he talked about hygiene. He

said after using the toilet, you must wash your hands properly. These are all responsible for typhoid.

Abu: All these things cause typhoid?

Sullay: Yes.

Abu: Mariama?

Mariama: Yes Abu.

Abu: How did you contract typhoid?

Mariama: Myself I don’t really understand. Hmm … I remember. When I visited Aunty Sylvia, the environment was filthy and I drank the unclean water. I am sure I contracted typhoid when I drank the unclean water.

Abu: Alright, it is okay now that we know.

Mariama: Yes Abu.

Abu: We are going to be careful now.

Mariama: Okay Abu.

Abu: Alright.

{Music playing}

Group A - Episode 3

My people, listen to this drama! With the Freetong Players International, Focus 1000 and the Karolinska institute in Sweden, we bring to you…

Episode three

Mariama: Thanks to God, I am feeling better now. However, I am only feeling weakness.

Abu: You seem to be feeling better now. Because now you can eat. You look okay now.

Mariama: I must eat Abu. What I went through, I do not want any member in my family to go through that. Because I nearly died.

Abu: I think we should fix a bed net.

Mariama: I am not sure. I think we should call the Doctor

Abu: Do you have the Doctor’s telephone number?

Mariama: Yes I have his number

Abu: Call the doctor.

{Phone ringing}

Mariama: Hello, hello?

Doctor: Who's on the line?

Mariama: Yes Doctor its Mariama

Doctor: How are you feeling now?

Mariama: I am feeling okay now, thank God

Mariama: Doctor to avoid contracting typhoid disease, are we to use bed net?

Doctor: No, No, No, Mariama, bed nets are meant for the prevention of mosquito bites, so that you don't get malaria. For typhoid, make sure you drink clean and pure water. And make sure you cook your food properly and make sure that when you come from the toilet, you wash your hands, so you have good hygiene. With that, you won't get typhoid.

Mariama: Ok doctor, thank you.

Doctor: you're welcome

Mariama: Ok bye bye.

{Music playing}

Group A – Episode 4

[Music]

My people, listen to this drama! With the Freetong Players International, Focus 1000 and the Karolinska institute in Sweden, we bring to you…

Episode four

[Phone rings] Hello? Hello Mariama, how are you doing?

Mariama: Yes Sister I am doing fine, and you?

Sister: Mariama it's been a while, what's going on?

Mariama: hmm it's a long story, I was sick and my husband took me to the health facility for treatment.

[Music, introducing flashbacks to previous episodes]

Man: Nurse?

Nurse: Yes Sir

Man: Nurse good morning. I brought my wife for treatment

Nurse: Madam good morning, what's your name and how are you feeling?

Mariama: My name is Mariama. Nurse, I am feeling weak, my stomach is aching and I have a fever.

Nurse: Your wife is having typhoid-malaria

Man: I said it

Mariama: [SHIVERING]

Man: That's why I decided to take her to the hospital for treatment

Head nurse: Nurse I heard you saying about typhoid-malaria. There is nothing like typhoid-malaria. There is a clear cut distinction between typhoid and malaria and they have different treatments.

Head nurse: Have you done a test?

Nurse: No Ma'am

Head nurse: Okay please go and do a test first

Nurse: Okay ma'am we will carry out the test

Head nurse: Do it now

Nurse injects patients and she shouted [woaaa]

Head nurse: Do you see, the patients does not have malaria and all the signs and symptoms are from typhoid. Therefore, she should be treated for typhoid.

[Back with her sister on phone]

Mariama: After realizing that I had typhoid, they treated me immediately. I didn't really know the cause of typhoid sister

[Introducing the next flashback, Someone knocks on the door bang bang]

Doctor: Who is knocking on my door?

Sullay: It's me Doc Sullay your friend

Doc: Eh Sullay please come in

Sullay: Doc it's been a while. I am here to ask you a question.

Doc: Yes, you may proceed

Sullay: I would like to know the cause of typhoid, it is caused by mosquitoes or what?

Doc: No, No, No, Sullay, mosquito bites is the not the cause of typhoid, it is caused by consumption of uncleaned water, uncooked food, unhygienic behaviour as not washing hands before and after using the toilets. That's all.

Sullay: Is that all?

Doc: Yes, that's all I have to say.

Sullay: Okay Doctor, thank you very much.

[Sullay returns home]

Sullay: Brother, aunty I am just coming from the Doctor

Sullay: The Doctor said that typhoid is caused by uncooked food, unclean water and thirdly, he mentioned that unhygienic attitudes will cause typhoid. He advised that we should wash our hands when we want to eat or use the toilet. He said that mosquito bites are not the cause of typhoid at all.

Family: Hmmm

[Mariama back to her sister on the phone]

Mariama: Sister, those were the measures I took to regain my health and I am fully aware of the things to do not to get typhoid.

Sister: Okay Mariama I have heard you and will abide to what you have said. I will make sure I abide to the rules and regulations regarding typhoid, so me and my family stays safe. Extend my sincere greetings to everyone

Sister: Alright Mariama bye

[Music continue playing till it fades out]

Intervention Group B: Avoiding Misinformation

Group B – Episode 1

[Music playing]

My people, listen to this drama! With the Freetong Players International, Focus1000 and the Karolinska institute in Sweden, we bring to you…

Episode 1

Abu: Mariama how are you feeling now?

Mariama: My stomach hurts, I am feeling weak and I have a fever. My sister is getting married soon and I don't know if I will have the chance to attend [shivering]

Abu: It's been five days since you've been shivering, I think you have to visit the hospital to know the cause of your illness

Mariama: I am unable to go the hospital

Abu: Okay I will accompany you to the hospital

Mariama: Okay

[Music playing]

AT THE HOSPITAL

Abu: Good morning nurse

Nurse: Yes sir, good morning

Abu: I came with my wife for check ups

Nurse: Madam, good morning

Mariama: Yes, nurse good morning

Nurse: How are you feeling?

Mariama: I am feeling weak, my stomach is aching

Nurse: Typhoid is disturbing her

Abu: Typhoid?

Nurse: Yes, she is suffering from typhoid

Abu: Typhoid?

Nurse: Of course, she has typhoid

Abu: Nurse I am not convinced. My wife is not only suffering from typhoid, I think there are other diseases.

Nurse: No, my brother, from your explanation, all signs and symptoms you have highlighted are signs that are attributed to typhoid diseases. People suffering from typhoid get sick and weak. Don't think typhoid won't make someone sick, it does.

Abu: Nurse, I insist, my wife is having another disease, it's not only typhoid disease.

Nurse: Okay!

Head nurse: Nurse, what's going on here?

Nurse: This man came to me with his wife and he told me that his wife feeing weak, she had stomach ache and I told him that his wife had typhoid and he said that his wife had another disease. This is the scenario.

Head nurse: No, she has typhoid. Typhoid usually comes all by itself and when you contract typhoid disease, you will begin to feel unwell and feel sick

Nurse: Exactly

Head nurse: Typhoid is treated with antibiotic drugs

Abu: Yes Sister

Abu: Don't worry okay, things will get better. Let's go home

[Music playing]

Mariama: Abu do you think I will get well soon and make it to my sister's wedding?

Abu: Yes, you will surely make it. Please continue with the drugs given to you at the facility

Mariama: Okay Abu

[Music playing]

Group B – Episode 2

{Music playing}

My people, listen to this drama! With the Freetong Players International, FOCUS 1000 and the Karolinska Institute in Sweden, we bring to you...

Episode Two

{Phone ringing}

Woman: Hello Mariama.

Mariama: Yes sister. How are you doing?

Woman: I am doing fine. I am here to check my wedding dress and I decided to call you to ask about your sickness.

Mariama: Sister, I went to the hospital and I was diagnosed with typhoid. Presently I am on treatment.

Woman: Mariama?

Mariama: Yes sister.

Woman: How did you get typhoid?

Mariama: My sister I don't really know.

Woman: Alright Mariama, I will pray for you to get better because I want to see you at my wedding.

Mariama: By God's grace.

Woman: Alright Mariama. Take care of yourself. Take it easy.

Mariama: Okay sister. Abu, Abu?

Abu: Yes Mariama.

Mariama: How do you get typhoid?

Abu: Mariama I don't know but let us ask Sullay.

Mariama: Okay Abu.

Abu: Let me ask him. Sullay?

Sullay: Yes brother?

Abu: Sullay come here!

Sullay: Yes brother.

Abu: How can a person get typhoid?

Sullay: Brother, I don't know. But I have a friend who is a well-known doctor. I will meet and ask him. He will explain exactly how typhoid is contracted.

Abu and Mariama: Okay. Alright.

{Music playing}

{Door knocking}

Doctor: Who is it?

Sullay: Doctor it is Sullay, your friend.

Doctor: Come in, come in. Long time, Sullay!

Sullay: Doctor I am here to see you. I have a question to ask you.

Doctor: Go ahead Sullay.

Sullay: How is typhoid contracted?

Doctor: Sullay, people get typhoid through contaminated water that is unclean water and also make sure you cook food properly and after using the toilet, wash your hands before touching food.

Sullay: Doctor is that all?

Doctor: That is all.

Sullay: Okay doctor, thank you.

Doctor: You are welcome.

Sullay: We shall meet again.

Doctor: Alright.

{Music playing}

Sullay: Brother and aunty I am back.

Mariama: Yes.

Abu: Sullay what did the doctor said?

Sullay: The doctor said a person can contract typhoid through drinking contaminated water or unclean water, uncooked food and also not washing your hands after using the toilet. Do you understand? That is what the doctor told me.

Abu: Mariama, how did you contract typhoid?

Mariama: Abu, I don't really understand … okay I remember when I went to Aunty Sylvia. The environment was not clean at all and the water Aunty Sylvia gave me to drink was unclean. That is how I contracted typhoid.

Abu: Okay. But you are on treatment now.

Mariama: Yes Abu.

Abu: I will make sure that will never happen again. Okay.

Mariama: Okay Abu.

{Music playing}

Group B – Episode 3

{Music playing}

My people, listen to this drama! With the Freetong Players International, FOCUS 1000 and the Karolinska Institute in Sweden, we bring to you...

Episode Three

Mariama: Thanks to God, I am feeling better now. However, I am still feeling weak.

Abu: You seem to be feeling better now. Because now you can eat. You look okay now.

Mariama: I must eat Abu. What I went through, I do not want any member in my family to go through that. Because I nearly died.

Abu: Mariama?

Mariama: Yes Abu?

Abu: What are we going to do to avoid typhoid?

Mariama: I don't know. Let us call Sullay.

Abu: Sullay.

Sullay: Yes Sir.

Abu: Sullay.

Sullay: Yes brother.

Abu: Sullay, I want you to call doctor to advise us what we should do to avoid contracting typhoid.

Sullay: Okay.

{Phone ringing}

Doctor: Hello.

Sullay: Yes doctor this is Sullay.

Doctor: Sullay, how are you?

Sullay: Not bad. Doctor I want to ask you a question.

Doctor: Go ahead.

Sullay: Doctor what should we do to avoid getting typhoid.

Doctor: Sullay.

Sullay: Yes.

Doctor: Make sure you drink clean water, cook your food properly and always was your hand clean.

Sullay: Thank you doctor.

Doctor: Okay, you are welcome.

Mariama: Okay Sullay thank you.

Sullay: Okay aunty.

Mariama: Abu.

Abu: Yes.

Mariama: I am feeling okay but I still feel the weakness. However, I am sure that I will go to my sister wedding.

Abu: That is true, you will go to sister wedding.

Mariama: Okay Abu.

Abu: You will go.

{Music playing}

Group B – Episode 4

{Music playing}

My people, listen to this drama! With the Freetong Players International, FOCUS 1000 and the Karolinska Institute in Sweden, we bring to you…

Episode Four

{At the wedding}

Mariama: Sister, congratulations. I am happy for you.

Sister: Thank you Mariama. I appreciate that you attended my wedding. Thank you my sister.

Mariama: My sister it wasn't easy what I went through.

Mariama: I have learned about typhoid. I learned that person can get typhoid when you drink contaminated water, eat contaminated food like uncooked food and live in a filthy environment. My sister. I also learned that to avoid typhoid, you should drink clean water, cook the food properly and wash our hands always.

First woman: Alright Mariama, let us celebrate.

{Music playing}
