# Supplementary material for: Debunking highly prevalent health misinformation using audio dramas delivered by WhatsApp: evidence from a randomised controlled trial in Sierra Leone
Source: BMJ Glob Health. 2021 Nov 9;6(11):e006954. doi: 10.1136/bmjgh-2021-006954 (PMC8578963; doi:10.1136/bmjgh-2021-006954)
Supplement: Supplementary data [file bmjgh-2021-006954supp002.pdf]

Study Protocol Contagious Misinformation Trial

Karolinska Institutet, Sweden
Maike Winters
Helena Nordenstedt
Helle Mölsted Alvesson
Zangin Zeebari
Carl Johan Sundberg
Mohamed F. Jalloh

FOCUS1000, Sierra Leone
Paul Sengeh
Mohammad B. Jalloh
Nance Webber
Samuel Abu Pratt
James Sengeh

COMAHS, Sierra Leone
Bailah Leigh

New York University, USA
Ben Oppenheim

Version 10: September, 2019

Table of Contents

Background & Rationale ***2***
Contagious Misinformation Trial ***3***
Typhoid-malaria 3
Research Questions ***4***
PICOT ***5***
Population 5
Intervention 5
Comparator 6
Outcome 7
Target 8
Sample Size, Sampling & Recruitment ***8***
Randomization ***10***
Data collection ***10***
Baseline survey 10
Follow-up survey 10
Blinding 11
Timeline 11
Data Analysis Plan ***12***
Ethical Considerations ***13***
References ***14***

Background & Rationale

In a time that is dubbed the 'post-truth era', people around the world are regularly exposed to misinformation and fake news (1). Whereas misinformation has been around since mankind, social media and the increased media fractionation has created echo chambers and information silos that can potentially amplify misinformation (1–3). This is problematic, as misinformation can lead to misconceptions, which might in turn influence knowledge and behaviour (4).

One of the most well-known examples of how misinformation influences behaviour concerns the (non-existing) link between the measles vaccine and autism. Following the fraudulent and retracted paper by Wakefield in the Lancet (5), online, very vocal anti-vaccination campaigners keep spreading false information and urge parents not to have their children immunized. In several European countries and in the USA, vaccine coverage has declined over the last years – which led to several measles outbreaks (6,7).
In other recent disease outbreaks, misinformation has played an important role as well. In the 2016 Zika outbreak for instance, a rumour went around that Zika was not the cause of microcephaly, but that is was a consequence of a larvicide (8). Similarly, blame was placed on vaccines distributed by the Brazilian government (9,10).

These kinds of misinformation and rumours can make people less likely to adhere to public health advice to curb an outbreak. In the world's largest outbreak of Ebola virus disease (EVD) in West Africa in 2014-2016, a widespread rumour dictated that through washing with salt and hot water, the body would be protected from the virus (11). Whereas the washing in itself might seem harmless, the belief that they were protected from the virus might have induced risky behaviours (11). In this outbreak, mass media such as radio, are believed to have played a role in disseminating incorrect information, which could have influenced societal perceptions of EVD and the likelihood for individuals to seek medical attention (12). A study looking at the public's exposure to different types of information channels during the EVD outbreak in Sierra Leone, found that especially radio was important in enhancing people's knowledge and protective behaviour, but was at the same time also associated with misconceptions and risk behaviour (13). Risk communication in a disease outbreak has the potential to save lives, but could also ravage communities, depending on the type of communication channel and the quality of the content.

It is therefore of vital importance to find ways to counter misinformation and misconceptions – especially in infectious disease outbreaks. Unfortunately, simply rejecting a piece of false information might have the opposite effect by actually enhancing the belief in the false information; the so-called backfire effect (14). This is especially true when the false information is in line with people's existing worldviews (15). For instance, a randomized trial in the USA using text messages with pro-vaccine information found that the effectiveness of the messages depended on the parental attitudes towards vaccination; among parents who were opposed to vaccination, the misconceptions were enhanced (16). Similar results have been reported elsewhere (17). A trial among staff of autism intervention centres in Australia aimed to decrease support for non-evidence-based autism therapies, while at the same time enhancing support for proven therapies (18). The study found that support for non-evidence-based therapies significantly decreased in the intervention group, whereas support for proven

therapies did not change. These effects were not sustained over time however, showing that more needs to be done to make the debunking effect stick long-term (18).

A meta-analysis investigating the effect size of different strategies of debunking misinformation found that certain types of refutations might actually enhance the belief in the wrong information (19). It was also found that most successful debunking strategies included detailed counterarguments (19). While providing a plausible alternative explanation to why information is wrong can reduce misconceptions, this effect is not always observed (19). Other suggestions that might yield positive results include not mentioning the wrong information at all (15). Furthermore, factors such as the credibility of the source, explanations in line with existing world views and social norms, giving a warning about misinformation coming up, showing graphical representations and salience of the corrective message all have the potential to enhance the chance of successful debunking (18).

A large part of the trials carried out so far, have been carried out in highly experimental settings, with relatively small sample sizes and included young, mostly female participants (college students) (19). Furthermore, most studies have focused on messages that were unfamiliar to the participants at the start of the trials. These studies started with a piece of misinformation which through interventions was tried to be countered (14,20–22). Not much is known about the effectiveness of correcting real-world misinformation; i.e. debunking misinformation that is already prevalent among the public. As described above, health-related misinformation can have grave consequences in infectious disease outbreaks. Whereas studies have reported the existence of misinformation and misconceptions around infectious diseases, to our knowledge no other study has studied the effectiveness of debunking strategies of misinformation about infectious diseases with a randomized trial.

Contagious Misinformation Trial

The Contagious Misinformation Trial (CMT) will be carried out in Freetown, the capital of Sierra Leone. Previous studies by the research team were set in Sierra Leone, using data from the EVD outbreak in 2014-2016 and were described above (11,13). For the CMT, misinformation about different infectious diseases were explored. The initial idea to try to counter lingering misinformation about Ebola was dismissed, as the disease is still a very sensitive topic in Sierra Leone. By investigating and spreading messages about Ebola, there is a risk that the public will mistake the renewed interest in the disease for a recurrence of an EVD outbreak.

Typhoid-malaria

Another disease that was found to be prone to misinformation was typhoid. The disease spreads through contaminated food and water and can be transmitted between people through the oral-faecal route. Typhoid can be successfully treated with antibiotics, however multidrug-resistant typhoid is increasingly common in West Africa (23). Symptoms of typhoid, such as high fevers, are similar to malaria. The estimated prevalence of typhoid in Sierra Leone is 0.01% (24).

In Sierra Leone, people tend to put typhoid and malaria together, often calling typhoid 'typhoid-malaria' (25,26). Given the believed similarity to malaria, many people think that

typhoid is caused by mosquitoes as well (in addition to contaminated food and water). People commonly think that typhoid and malaria come together: usually you get malaria first, and then typhoid comes on top of it.

Typhoid is ideally diagnosed through blood culture. Unfortunately, in Sierra Leone, there is only one hospital that has the equipment to do this, but even there, resources are not sufficient to carry out the test (personal communication at Connaught Hospital Freetown). Instead, the Widal test is commonly used across the country to diagnose typhoid. This diagnostic test has reportedly low sensitivity, specificity and positive predictive value (27,28). It is suggested that the Widal test cross-reacts with malaria antigens, making the Widal test more likely to be positive among malaria patients (29). True co-infection of typhoid and malaria rarely happens (30,31). In reality however, with the poor performance of the Widal test, patients frequently get to hear in the health centers in Sierra Leone that they have 'typhoid-malaria'.

The belief in this misinformation around typhoid seems to be prevalent, with 70-80% of respondents in our small survey responding that typhoid is caused by mosquitoes. Given the low number of doctors and health centres in Sierra Leone, many people visit a traditional healer or a local pharmacy to self-medicate. Thinking that they might have typhoid, people take antibiotics and antimalarials – both drugs are prone to become resistant. Countering the misinformation around typhoid can therefore not only improve health-related knowledge, but can potentially also have an effect on drug-taking habits.

Two pieces of misinformation will be targeted in the CMT:

- The belief that typhoid is caused by mosquitoes
- The belief that typhoid can only come together with malaria

In the CMT, we will evaluate whether our intervention of a series of audio messages in the form of an audio drama is effective in countering misinformation.

Research Questions

Main research question:

Does the proposed intervention of audio messages reduce the belief in misinformation about typhoid compared to a control group among adults in urban Freetown in Sierra Leone, using an intention-to-treat analysis?

Secondary research questions:

1. Does the proposed intervention of audio messages prevent a 'backfire' effect, whereby the belief in misinformation is enhanced?
2. Does the proposed intervention of audio messages reduce the belief in misinformation about typhoid compared to a control group among adults in urban Freetown in Sierra Leone, using an 'as treated'-analysis?
3. Does the proposed intervention of audio messages increase the knowledge about preventive methods for typhoid compared to a control group?
4. Does the proposed intervention lead to more health-related discussions with family and friends compared to a control group?

5. Is the proposed intervention successful in reducing the belief in misinformation and increasing the belief in the correct information about typhoid when administered in an alternative way?

PICOT

Population

Adults aged 18 and older in urban Freetown, with fluency in Krio, in possession of a mobile phone that has WhatsApp. Systematic random sampling will be used to select sections in urban Freetown. The Housing Census List of Enumeration Areas will function as a sampling frame, comprising 64 Sections in the Western Urban area. Within the selected sections, a modified random walk method will be used to select households with a predetermined skip interval. Exclusion criteria: Deafness, as one needs to be able to hear the audio messages.

Intervention

There are two intervention groups in the CMT: The Plausible Alternative group and the Avoiding Misinformation group (see figure 1). In line with previous research, offering a plausible alternative, i.e. explaining why the misinformation is wrong, has been shown to have a higher success rate of countering misinformation than simple rejections, that can backfire and unintentionally reinforce the misinformation (14). Another, slightly less explored strategy to counter misinformation is through avoiding mentioning the wrong information entirely (15).

In the **Plausible Alternative group,** we will explicitly mention the wrong information. This is then followed by an explanation of why this is not true and providing the alternative, correct information.

The messages in the **Avoiding Misinformation group** will instead solely focus on the correct information about that typhoid; the causes, as well as ways to prevent infection. Any mentioning of the existing misinformation around typhoid will be avoided.

<u>Core elements</u>

Consultations with media experts in Sierra Leone and published literature highlight the need to have a trusted messenger deliver the information (32). Furthermore, repeated exposure to messages is vital to increase the chances of successful debunking. Some studies thus far have been limited in this approach, by only letting participants be confronted to debunking messages at one timepoint (20–22). In the CMT we will have 4 messages (i.e. 4 episodes) per intervention group, of around 4-5 minutes each. The episodes will be sent to the participants every 7 days.

<u>Content of the audio dramas</u>

The audio messages will be in the form of an audio drama with a clear storyline, making the messages easy to follow and creating an emotional connection with the audience. The Sierra Leonean actor's group Freetong Players will develop the audio dramas. They are a well-known and trusted group of actors in Sierra Leone, and highly experienced in making audio dramas. To ensure that the message comes from a trusted authority, there is a statement in each

episode that the message was developed by FOCUS1000 and Karolinska Institutet. The format will be the same in the two intervention groups, but the content will differ. The core message of each episode in the two intervention groups are described in table 1. The storylines for the two audio dramas can be found in Appendix A.

Table 1. Core messages per episode per intervention group

Episode	Group 1: Plausible Alternative	Group 2: Avoiding Misinformation
1	People think there is a disease called typhoid-malaria, but these are two different diseases	You can get typhoid by itself, without having other diseases
2	Typhoid is not caused by mosquitoes, only by contaminated water and food	Typhoid is only caused by contaminated water and food
3	People think sleeping under a bed net helps to prevent typhoid, but actually good hygiene, drinking treated water and cooking food properly help prevent typhoid	Prevent yourself from getting typhoid by cooking your food properly and drinking only treated water
4	Repetition of messages of episodes 1-3	Repetition of messages in episodes 1-3

<u>Administration of the intervention</u>

Participants in the CMT will receive the four episodes on their phone, through WhatsApp. The first episode will be sent to the participants on Monday October 21st, 2019. Every 7th day after that, the following episode will be sent on WhatsApp, using the Broadcast function on WhatsApp whereby one sender can send messages to up to 256 recipients. Recipients will receive the message in an individual chat, so they don't see who else received the message. Before receiving the audio message, the participant receives 10.000Le data credit, to ensure that the audio message can be received. The intervention will last for 4 weeks.

WhatsApp is commonly used in Sierra Leone. The advantage of this platform is that we can see whether the message has arrived and (for the large majority) whether it has been seen – we don't know whether it has been listened to however. Restricting the sample to only those that have WhatsApp means in practice that we will probably exclude the poorest part of the population, as they are unlikely to have a phone that is suitable for WhatsApp. This also means that the educational level of our participants will likely be higher than the average educational attainment of the Sierra Leonean population. To compare whether the audio dramas work irrespective of WhatsApp, we will recruit 60 people who don't have WhatsApp. They will be called every week and listen on the phone to the episodes. They will be randomized so that 30 of them will listen to the episodes from intervention group 1 (Plausible Alternative) the other 30 to the episodes of intervention group 2 (Avoiding Misinformation). Similar to the WhatsApp groups, these 60 people receive the baseline and follow-up surveys. The two Non-WhatsApp groups will be used to answer secondary research question 5.

Comparator

In the primary analysis, the two interventions will be compared to a control group. To keep participants engaged in the trial, and to avoid contaminating the control group with messages about typhoid or malaria, messages to the control group will be about exclusive breastfeeding. Participants in the control group will receive 2 messages, which will be sent at the same time as episode 1 and 3 are to be sent to the intervention groups. Similar to the intervention

groups, the participants in the control group will receive 10.000Le data credit directly before receiving the messages.

Outcome

Primary outcomes

The primary outcomes of the CMT are the reduced beliefs in two pieces of misinformation about typhoid:

1. Typhoid is caused by mosquitoes
2. Typhoid can only come together with malaria

These beliefs will be captured in the baseline and follow-up surveys with 'yes/no' questions. The primary analysis will be an intention-to-treat analysis.

Secondary outcomes

1. We will test a potential 'backfire effect', whereby our intervention unintentionally might have increased the belief in the misinformation.
2. We will carry out an 'as-treated-analysis' for the primary outcomes and the potential backfire effect. This will be captured through questions in the follow-up survey asking about the content of the intervention messages. Only the participants who accurately remember the content will be included in this analysis.
3. Knowledge about preventive methods for typhoid will be captured with the question: 'Can you name up to three ways how you can prevent yourself from getting typhoid?'. This will be asked as an open question; the data collector ticks the appropriate boxes. Based on how many correct / incorrect answers the participant gives, a score will be created that can vary between -3 and +3.
4. To estimate whether the invention leads to more health-related discussions with family and friends, we ask 'How often do you discuss health issues with family or friends?' in the baseline and follow-up. In the follow-up, there will be an additional question: 'Did you discuss the audio fragments with friends and/or family?' to which people can answer yes or no.
5. To understand whether a reduction in the belief in misinformation and increase in the belief in the correct information is also achieved without using WhatsApp, 60 people with no WhatsApp will be recruited. We will carry out analyses to test the primary outcome, comparing the Non-WhatsApp groups with the control group, and with the WhatsApp intervention groups.
6. Self-efficacy will be tested in the follow-up survey only, through 3 questions based on 3 preventive method, e.g. 'how confident do you feel in your abilities to cook food properly?' We will evaluate whether there is a difference between the intervention groups and control group in people's self-efficacy.
7. To understand whether the interventions had an effect on risk perception and preventive behaviours of typhoid compared to the control group, we will evaluate the questions about risk perception and typhoid actions. The hypotheses put forward by Brewer et al., 2004 (33) will be tested using these questions. This analysis will likely be published in a separate paper.
8. People might feel they have learned something from the audio dramas; to test whether subjective and objective learning match, we associate the outcomes of the question 'do you feel that you learned something from the audio dramas?' with the scores on the primary outcomes.

Target

This is a superiority trial, to find out if any of the intervention groups is better in achieving the primary outcome than the comparator.

Flowchart of trial

Figure 1. Flowchart of the Contagious Misinformation Trial

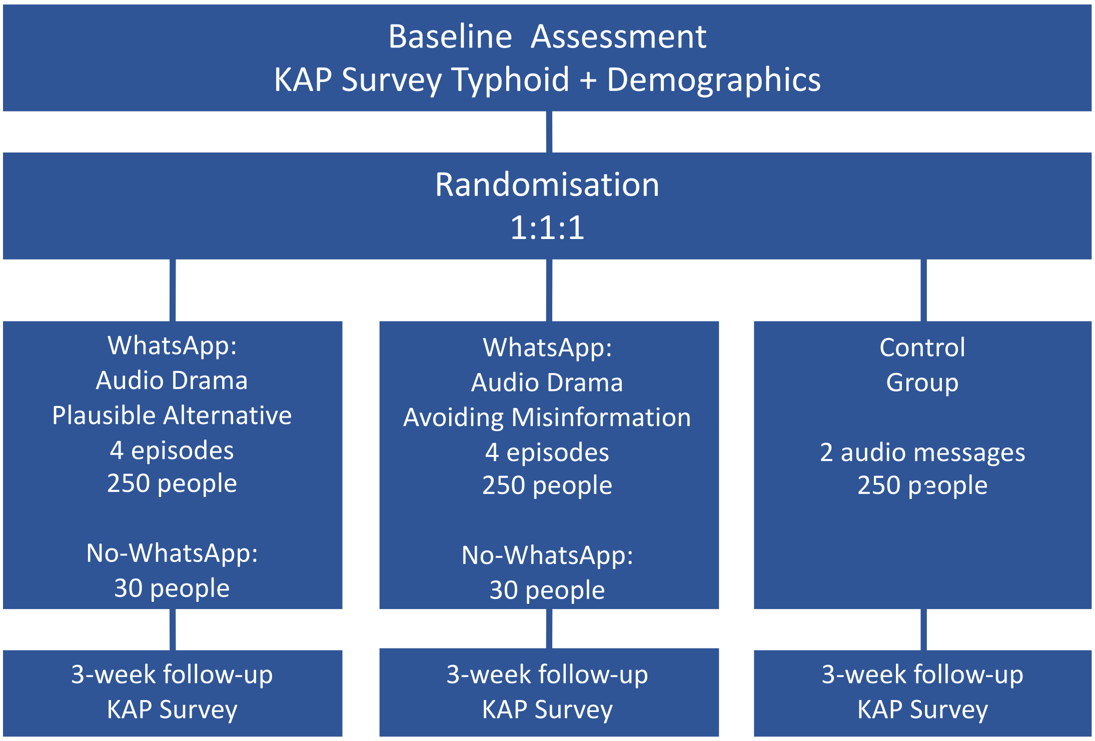

Sample Size, Sampling & Recruitment

Sample Size

Sierra Leone comprises 4 regions, with 14 districts. Freetown is part of the Western Urban district, with about 1.5 million inhabitants. Western Urban is further divided into 64 sections (see figure on next page), which are comprised of 2139 Enumeration Areas. Enumeration Areas each contain about 80-110 households.

For the Contagious Misinformation Trial (CMT), we will randomly select 20 of the 64 sections in the Western Urban district. The 64 sections vary in the number of households they contain: the largest has over 6000 households and the smallest around 600. Therefore, the random selection of the cluster will be weighted proportionate to size, so that the clusters with the largest number of households have more chance to be included than smaller clusters. In Excel, the 64 sections are sorted according to size, starting with the smallest cluster. Next, we calculate the cumulative percentage of the sections. Using a variable that creates 21 random numbers between 0 and 1, the sections that are closest to the random numbers in terms of

the cumulative percentage will be selected. If through this method a section will be randomly selected more than once, the system will automatically pick a new number until there are 21 exclusive sections selected.

During the recruitment and data collection period (8 days for baseline), we will send 3 teams of 4 data collectors and 1 supervisor to one section every day. The enumerators have the task to recruit 32 people in that section on that day. The team will start in the approximate middle of the section and will drop a pen there. The first data collector will walk in the direction of the tip of the pen, the second data collector will go in a direction about 90 degrees from the first data collector, data collector 3 in a 180 degrees angle, etc until all 4 enumerators have found their direction. All data collectors will use a skip interval of 15 houses. The first interview of the day will be with a woman, the second with a man, the third with a woman etc. In case there is nobody home, or nobody eligible is available, a house one skip interval away will be approached. Data collectors will make notes how many houses they approached and how many declined to participate and this will be reported back to the research team. With 12 data collectors and 3 supervisors, we will cover 3 sections per day, recruiting 96 people per day. The supervisors will be supervising only in the first 2 days of data collection; they will visit the enumerators, join their recruitment and survey administration tasks and provide feedback. From day 3, the supervisors will recruit and collect data as well. Supervisors will focus on recruitment of 4 people per day who do not have WhatsApp. The enumerators will reached the target sample size of 750 in 7 days, the 3 supervisors will reach the target of 60 people with no WhatsApp in 5 days.

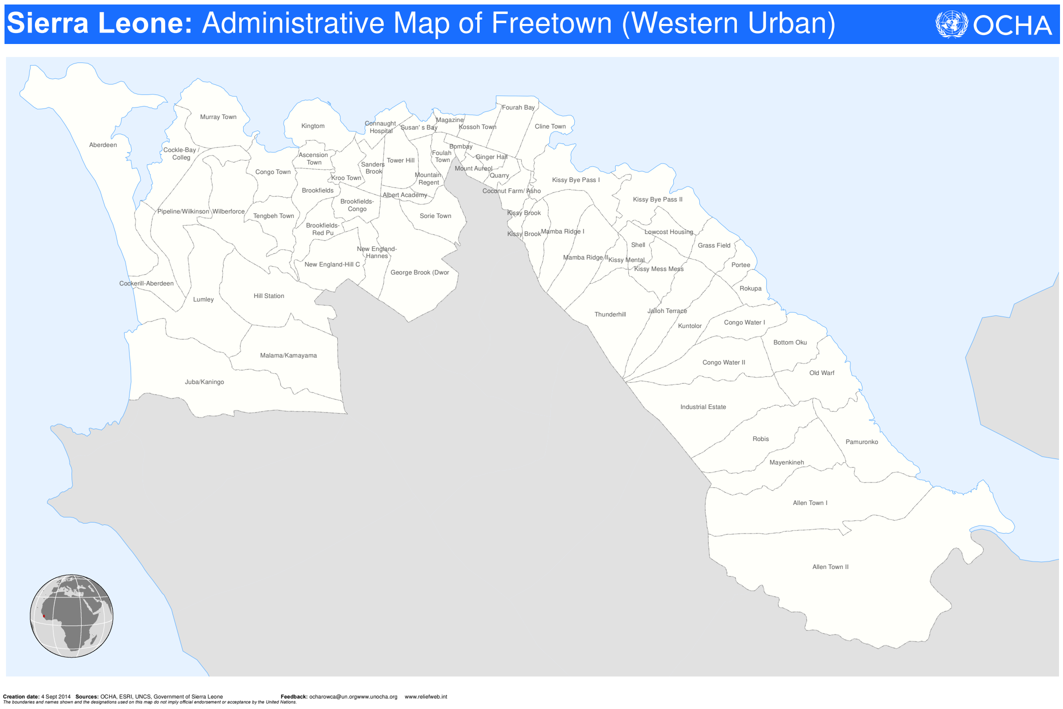

From: https://www.humanitarianresponse.info/sites/www.humanitarianresponse.info/files/Freetown_A1_L_140904_AdminMap.pdf
Other useful map: https://www.openstreetmap.org/#map=15/8.4333/-13.1640

Recruitment
Participants will be recruited based on the following inclusion criteria:

- Adults (18 years or older)
- In possession of a mobile phone that has WhatsApp
- Fluency in Krio
- Good hearing ability

If yes, the potential participant will be extensively briefed on what it means to participate in the trial, guided by an information sheet (Appendix B). When the participant agrees to take part in the trial, the data collector will also explain how WhatsApp audio messages are to be received and listened to. We ask the participant to program the phone number from which the audio messages in the participant's phone, so that we ensure that audio messages can be received. The information sheet will be given to the participants, with contact details of the trial staff. If the participant gives consent, the baseline survey will be administered. In case the participant is illiterate, verbal consent will be recorded.

Randomization

After the required sample size has been reached, all data will be downloaded in an Excel file and split into the WhatsApp groups (i.e. 750 participants) and the Non WhatsApp groups (60 participants) Randomization will be done using already programmed code in Excel that randomly assigns each of the participants in one of the three groups (i.e. 2 intervention groups, 1 control group). The code will also ensure that each group will end up with 250 participants. The same code, but adjusted to 2 groups will be applied to the Non WhatsApp spreadsheet.

Data collection

Baseline survey

Directly after informed consent has been given by the participant, the baseline survey will be administered. This survey contains questions about basic demographics (age, sex, educational level, religion etc.), after which questions about typhoid and malaria are asked. The data collector asks the questions in person and directly enters the answers on the data capturing tablet. The baseline survey was pilot tested in May 2019 in Freetown. The latest version of the survey is attached in Appendix C.

Follow-up survey

At baseline, phone numbers and addresses of participants will be collected. A few days before the follow-up survey is to be administered, the participant will be called / WhatsApp'ed, to schedule a time for the follow-up survey. Similar to the baseline data collection, teams of 4 enumerators and 1 supervisor will visit every day a different section to do all follow-up surveys. The same teams will go to the same sections as much as possible, so that we ensure that the households can be easily traced back. If the participant is not home, the participant will be called and an alternative time will be arranged. We try as much as possible to do all follow-up surveys in the same section on the same day, but if this is not possible, the interview will be conducted on one of the last days. If the participant can still not be met in person, we will conduct the survey over the phone.

The survey administered at follow-up (around 3 weeks after the last episode has been sent out) is similar to the baseline survey, but has two important differences:

1. It does not contain the questions about demographics.
2. It contains extra questions regarding the content of the messages, to capture whether participants have actually listened to it.

After completion of the follow-up survey, the participant will receive the final 10.000Le for data top up. The participant will also receive an information sheet explaining typhoid and malaria, so that all participants (also the control group) will have the correct information at the end of the study.

<u>FOCUS1000</u>
Our main partner for the data collection is the Sierra Leonean non-governmental organization FOCUS1000. During the EVD outbreak in 2014-2016, they initiated the planning and data collection of four Knowledge, Attitude and Practice Surveys. This data was used to inform the ongoing outbreak response and were later the basis of several scientific publications.

Given their experience and expertise in data collection in Sierra Leone, FOCUS1000 will take the lead in data collection. They will oversee the data collection process, recruit suitable data collectors and supervisors from their pool of experienced data collectors, loan tablets for data capturing and they will administer the surveys at baseline and follow-up. The data collectors should be fluent in English and Krio. The data capturing software 'Kobo Collect' will be used on the tablets. The linguistics department of the University of Freetown will translate the survey from English to Krio, both in a written version and in a spoken version.

Blinding

Participants are blinded to the assignment of the group. As all groups (also the control group) will receive audio messages, participants will not know whether they were in the intervention or in the control group. Data collectors are to a large degree blinded to the assignment of the groups. The follow-up survey will contain questions about the content of the audio drama (which will be used for the as-treated analysis), answering those questions will reveal the group the participant was in. These questions will be asked last, so that the data collector is blinded up to those questions. The data analysis team at Karolinska Institutet will only receive the anonymized dataset.

Timeline

The data collection will begin in October 2019. Before that, in September, 12 data collectors and 3 supervisors will be recruited by FOCUS1000. Training will be organized by both KI and FOCUS100 in Freetown in the first week of October and is expected to last 3 working days. Recruitment and administration of the baseline survey will take place between 7-15 October. The intervention will start on October 21st and will last 4 weeks. The follow-up survey will be administered 3-4 weeks after the last episode of the audio dramas has been sent. There will be another 2-day training for the data collectors before the start of the follow-up survey in early December.

Key dates in 2019:

Oct 2-4: Training of data collectors at FOCUS1000

Oct 7-15:	Recruitment of participants and administration of baseline survey
Oct 21:	Episode 1 to the two intervention groups<br>Message 1 to the control group
Oct 28:	Episode 2 to the two intervention groups
Nov 4:	Episode 3 to the two intervention groups<br>Message 2 to the control group
Nov 11:	Episode 4 to the two intervention groups
Nov 28-29:	Training of data collectors at FOCUS1000
Dec 2-14:	Administration of the follow-up survey

Data Analysis Plan

A core team comprising of a statistician, an Associate Professor, a PhD student and a social scientist, will carry out the statistical analysis, using STATA v.15. Before any analysis is done, the team will determine the extent of missing values in the dataset. Any patterns that are found in missing values will be reported. All models, statistical tests and table shells are detailed in a separate Statistical Analysis Plan.

Baseline characteristics

Baseline characteristics of the clusters, and of individuals will be calculated and described in two tables, stratified by trial arm.

Primary analysis

The primary outcomes of this trial are the reduced beliefs in the two main pieces of misinformation about typhoid. Both outcomes are binary, based on ‘yes/no’ questions. The proportions of participants believing in the misinformation in the intervention groups and control group will be summarized both at baseline and at follow-up. In this intention-to-treat analysis, the primary analysis will be a logistic regression test to determine whether there was a difference between the intervention groups and control group follow-up. Several models will be fitted, adjusting for covariates, including age, sex, educational level and religion. Results will be presented in a table, together with p-values and 95% Confidence Intervals to demonstrate whether results are statistically significant.

Secondary analyses

1. The backfire effect will be analysed through ordinal logistic regression models, whereby the outcome has three levels: increased belief in misinformation, no change in belief in misinformation, decreased belief in misinformation. Models will be adjusted for covariates.
2. The ‘as-treated-analysis’, will be carried out among the respondent who accurately remember the messages. This will be evaluated using the memory questions of the follow-up survey. Participants answering at least those questions correctly will be included in the analysis. The as-treated-analysis will be done for the primary outcomes as well as the backfire effect.
3. A score will be created for knowledge about preventive methods for typhoid, reflecting how many correct or incorrect preventive methods the participants names. Ordinal logistic regression analysis will determine whether there is a difference between intervention and control groups knowledge about preventive methods.

4. A logistic regression analysis will be carried out to determine whether there is a difference in health-related discussions among intervention and control groups.
5. The primary analysis will be carried out comparing the non-WhatsApp groups with the control group and with the WhatsApp groups.
6. The effect of the intervention on self-efficacy around three preventive methods will be analysed using ordinal logistic regression models.
7. The influence of the intervention on risk perception and preventive practices and associations between those two will be tested using Brewer et al (2004)'s three hypotheses
8. A Chi-square analysis will be carried out to determine whether participants who felt that they learned from the audio dramas in reality also scored better on the primary outcomes.

Post hoc analysis

If the analyses yield significant results, post hoc analyses will be carried out to determine if there are statistically significant differences between the two different intervention groups.

Ethical Considerations

Potential benefits and the generation of evidence should never come at a cost of participants of medical research. This research project will adhere to the premises of respect of persons, beneficence and justice.

In this randomized controlled trial, the research participants are the individuals to whom the interventions are administered. All participants will be extensively briefed on what it means to participate in the study and will not be further enrolled before an informed consent form is signed.

All information regarding the study, including contact details of field team members and the principal investigator will be provided in writing to all participants. They will further be ensured that they can drop out at any stage of the trial, without having to give a reason for doing so. Given the high illiteracy rates in Sierra Leone, it is likely that some of the participants won't be able to read the consent form. We will pursue the inclusion of illiterate or low-literate adults, provided that they meet the other inclusion criteria of using WhatsApp (which illiterate people tend to do, mainly for audio messages). In case of illiteracy, a field team member will read all information out loud and, if agreed, verbal consent will be given, which will be written down by the field team member and a witness.

Participating in the study is not expected to present any harm to participants. However, if participants feel they are exposed to any harm or risk, they are encouraged to contact the team members of the study. Contact details will be provided to all participants.

Ethical permission for this study was granted by the Sierra Leone Ethics and Scientific Review Committee on May 30th, 2019. The Swedish Ethical Review Authority in Stockholm has granted ethical permission: dnr 2018/1276-31.

References

1. Lewandowsky S, Ecker UKH, Cook J. Beyond Misinformation: Understanding and Coping with the "Post-Truth" Era. J Appl Res Mem Cogn [Internet]. 2017;6(4):353–69. Available from: http://dx.doi.org/10.1016/j.jarmac.2017.07.008
2. Prior M. Liberated Viewers, Polarized Voters - The Implications of Increased Media Choice for Democratic Politics. Good Soc. 2002;11(3):10–6.
3. Vosoughi S, Roy D, Aral S. The spread of true and false news online. Science. 2018;359(March):1146–51.
4. Southwell B, Thorson E, Sheble L. The Persistence and Peril of Misinformation. American Scientist. 2017. p. 1–11.
5. Wakefield A, Murch S, Anthony A, Linnell J, Casson D, Malik M, et al. RETRACTED: Ileal-lymphoid-nodular hyperplasia, non-specific colitis, and pervasive developmental disorder in children. Lancet. 1998;351:637–41.
6. Thornton J. Measles cases in Europe tripled from 2017 to 2018. BMJ. 2019;364(1634):doi: https://doi.org/10.1136/bmj.l634.
7. World Health Organization. Measles cases spike globally due to gaps in vaccination coverage [Internet]. 2018 [cited 2019 Apr 15]. Available from: https://www.who.int/news-room/detail/29-11-2018-measles-cases-spike-globally-due-to-gaps-in-vaccination-coverage
8. Navarro A. Larvicide Manufactured by Sumitomo, Not Zika Virus, True Cause Of Brazil's Microcephaly Outbreak: Doctors [Internet]. Tech Times. 2016 [cited 2019 Mar 10]. Available from: https://www.techtimes.com/articles/133548/20160214/monsanto-larvicide-not-zika-virus-true-cause-of-brazils-microcephaly-outbreak-doctors.htm
9. Worth K. As Brazil Confronts Zika, Vaccine Rumors Shape Percptions [Internet]. PBS. 2016 [cited 2019 Mar 10]. Available from: https://www.pbs.org/wgbh/frontline/article/as-brazil-confronts-zika-vaccine-rumors-shape-perceptions/
10. Bode L, Vraga EK. See Something, Say Something: Correction of Global Health Misinformation on Social Media. Health Commun [Internet]. 2018;33(9):1131–40. Available from: https://doi.org/10.1080/10410236.2017.1331312
11. Jalloh MF, Sengeh P, Monasch R, Jalloh MB, DeLuca N, Dyson M, et al. National survey of Ebola-related knowledge, attitudes and practices before the outbreak peak in Sierra Leone: August 2014. BMJ Glob Heal [Internet]. 2017;2(4):e000285. Available from: http://gh.bmj.com/lookup/doi/10.1136/bmjgh-2017-000285
12. World Health Organization. Ebola: Experimental therapies and rumoured remedies [Internet]. 2014. Available from: http://www.who.int/mediacentre/news/ebola/15-august-2014/en/
13. Winters M, Jalloh MF, Sengeh P, Jalloh MB, Conteh L, Bunnell R, et al. Risk Communication and Ebola-Specific Knowledge and Behavior during 2014–15 Outbreak, Sierra Leone. Emerg Infect Dis [Internet]. 2018;24(2):336–44. Available from: http://wwwnc.cdc.gov/eid/article/24/2/17-1028_article.htm
14. Lewandowsky S, Ecker UKH, Seifert CM, Schwarz N, Cook J. Misinformation and Its Correction: Continued Influence and Successful Debiasing. Psychol Sci Public Interes. 2012;13(3):106–31.
15. Cook J, Lewandowsky S. The debunking handbook. 2011.
16. Nyhan B, Reifler J, Richey S, Freed GL. Effective Messages in Vaccine Promotion : A Randomized Trial. Pediatrics. 2014;133(4):e835–42.
17. Pluviano S, Watt C, Sala S Della. Misinformation lingers in memory: Failure of three pro-vaccination strategies. PLoS One. 2017;12(7):1–15.
18. Paynter J, Luskin-Saxby S, Keen D, Fordyce K, Frost G, Imms C, et al. Evaluation of a template for countering misinformation — Real-world Autism treatment myth debunking. PLoS One. 2019;14(1):1–13.
19. Chan MS, Jones CR, Hall Jamieson K, Albarracin D. Debunking: A Meta-Analysis of the Psychological Efficacy of Messages Countering Misinformation. Psychol Sci. 2017;28(11):1531–46.

20. Ecker UKH, Lewandowsky S, Apai J. Terrorists brought down the plane !— No , actually it was a technical fault: Processing corrections of emotive information. Q J Exp Psychol. 2011;64(2):283–310.
21. Ecker UKH, Lewandowsky S, Fenton O, Martin K. Do people keep believing because they want to? Preexisting attitudes and the continued influence of misinformation. Mem Cogn. 2014;42:292–304.
22. Ecker UKH, Lewandowsky S, Cheung CSC, Mayberry MT. He did it! She did it! No, she did not! Mutiple causal explanations and the continued influence of misinformation. J Mem Lang. 2015;85:101–15.
23. Dyson ZA, Klemm EJ, Palmer S, Dougan G. Antibiotic Resistance and Typhoid. Clin Infect Dis. 2019;68(Suppl 2):S165-170.
24. Institute for Health Metrics and Evaluation. GBD Results Tool: prevalence of typhoid fever and malaria in Sierra Leone [Internet]. GBDx. 2021 [cited 2021 Jun 30]. Available from: http://ghdx.healthdata.org/gbd-results-tool
25. Kanu A. Typhoid-Malaria: The Evidence. Sierra Express Media [Internet]. 2012 Mar 3; Available from: http://sierraexpressmedia.com/?p=37721
26. Lebbie A, Wadsworth R, Saidu J, Bangura C. Predictors of Hypertension in a Population of Undergraduate Students in Sierra Leone. Int J Hypertens. 2017;1–10.
27. Andualem G, Abebe T, Kebede N, Gebre-Selassie S, Mihret A, Alemayehu H. A comparative study of Widal test with blood culture in the diagnosis of typhoid fever in febrile patients. BMC Res Notes. 2014;7(653):1–6.
28. Nsutebu F, Martins Ndumbe P, Koulla S. The increase in occurrence misuse of the Widal test ? of typhoid fever in Cameroon : overdiagnosis due to. Trans R Soc Trop Med Hyg. 2002;96:64–7.
29. Pradhan P. Coinfection of typhoid and malaria. J Med Lab Diagnosis. 2011;2(July):22–6.
30. Kargbo MS, Massaquoi LD, Samura SK. The Relative Prevalence of Typhoid and Malaria in Febrile Patients in Freetown , Sierra Leone. Open J Prev Med. 2014;4(May):338–46.
31. Mutua JM, Wang F, Vaidya NK. Mathematical Biosciences Modeling malaria and typhoid fever co-infection dynamics. Math Biosci [Internet]. 2015;264:128–44. Available from: http://dx.doi.org/10.1016/j.mbs.2015.03.014
32. Swire B, Ecker U. Misinformation and Its Correction. In: Southwell BG, Thorson EA, Sheble L, editors. Misinformation and Mass Audiences. Austin: University of Texas Press; 2018. p. 195–211.
33. Brewer NT, Weinstein ND, Cuite CL, Herrington JE. Risk Perceptions and Their Relation to Risk Behavior. Ann Behav Med. 2004;27(2):125–30.

Appendix A Storylines per intervention group

Group 1: Plausible Alternative

	Core message
Episode 1	Typhoid and malaria are 2 different diseases
Episode 2	Typhoid is not caused by mosquitoes
Episode 3	Preventive methods for typhoid
Episode 4	Recap of Episodes 1,2,3:

Episode 1:

- At home
- A woman (Mariama) is getting sicker every day (weakness, fever) since several days. Her husband (Abu) brings her to the health clinic
- In the health clinic, a nurse diagnoses the woman with typhoid-malaria, gets treatment Husband says – "I told you".
- Head nurse overhears the typhoid-malaria diagnosis and intervenes, orders the nurse to do a malaria test, which is negative
- Mariama gets diagnosed with typhoid
- The head nurse calls all the other nurses, and explains that people talk a lot about typhoid-malaria, but that this doesn't exist
- 'Typhoid is typhoid, malaria is malaria'
- End of episode tune

Episode 2:

- Mariama is getting better, but wonders how she got typhoid
  → Was is from mosquitoes?
- Mariam and Abu don't know, ask their neigbour Abu
- Abu knows a doctor, goes there to ask for clarifications
- Doctor explains that mosquitoes cause malaria, but tyyphoid is caused by other things: eating bad food, drinking bad water, bad hygiene
- Abu reports this back to Mariama and Abu
  → Mariama figures it out; she drank bad water at Aunt Sylvia's place 2 weeks ago
- End of episode tune

Episode 3:

- Mariama has recovered. But she never wants to experience typhoid again; she felt like she was almost dying.
- The couple discusses how they can prevent themselves from getting typhoid
  → Maybe by using a bed net?
- Abu calls doctor, who explains that bed net will help against malaria, not typhoid.
- To avoid typhoid, they should drink good water and wash hands and cook food properly

Episode 4 (RECAP of episodes 1-3)

- Recap of the 3 episodes, highlighting the core messages of each episode

Group 2: Avoiding Misinformation

	**Core message**
Episode 1	Typhoid diagnosis, it normally comes alone
Episode 2	Typhoid is caused by dirty water & food
Episode 3	Preventive methods for typhoid
Episode 4	Recap of Episodes 1,2,3:

Episode 1:
- A woman (Mariama) is getting sicker every day (weakness, fever) since several days. Her husband (Abu) brings her to the health clinic
- A nurse diagnoses the patient with typhoid and explains a bit about treatment
  - → Typhoid normally comes alone
- Head nurse overhears, and confirms
  - → Abu and Mariama are surprised, she is so sick, there must be more?
- Head nurse confirms that you can get very sick from typhoid
- Mariama worries that she can't go to her sister's wedding in 2 weeks
- (no mention of malaria or typhoid-malaria)

Episode 2:
- Mariama is getting better with the treatment
- They ask around to understand what caused typhoid
- Abu calls doctor, who confirms the correct information
  - → Doctor explains it is bad water (water no good for drink) and bad food
- Patient and husband try to figure out how the patient got infected
  - → Why did Mariama get sick and not the husband? (Remember 2 weeks ago when I was so thirsty at Aunty Silvia's place and I drank water outside her house that looked dirty?)
- No mention of mosquitoes
- Still anxious that she won't be fit enough for her sister's wedding

Episode 3:
- The patient has almost recovered. But she never wants to experience typhoid again; she felt like she was almost dying.
- The couple discusses how they can prevent themselves from getting typhoid
- Doctor: To avoid typhoid they should drink good water and wash hands and cook food properly

Episode 4 (RECAP of episodes 1-3)
- Mariama made it to her sister's wedding
- Tell her sister what she went through and what she learned on the way

Appendix B

Information for participants: The Information Na Pawa Study

Dear Sir/Madam,

You are asked to participate in a scientific study. Participating in this study is voluntary. Your participation requires your written consent. Before you decide whether you want to participate in this study, you will be given an explanation about what the study involves. Please read this information carefully and ask the investigator/enumerator for an explanation if you have any questions.

1. **General Information**

This study is carried out by FOCUS1000 in Freetown and Karolinska Institutet, a university based in Stockholm, Sweden. This study has been approved by the Sierra Leone Ethics and Scientific Review Committee and by the Swedish Ethical Authority.

2. **Purpose & background of the study**

The purpose of this study is to understand how people perceive information about health. It is important that the general public has a good understanding of diseases that can threaten their health, so that people know how to prevent and treat diseases.

3. **What participation involves & what is expected of you**

If you agree to participate in this study, the enumerator will administer a survey, asking questions about you and about certain health topics. People who participate in this study will be assigned to one of the three groups in this study. Depending on which group you will be randomly assigned to, you will receive between 2-4 audio messages on your WhatsApp. This starts on Monday October 21st; you will receive first 10.000Le data top up and that same day you will receive an audio message on your WhatsApp. We ask you to listen to all the audio messages you receive. Note that we can see whether you have listened to the messages. Please don't share the audio messages further in any way, we only want YOU to listen to them. You can discuss the content with family or friends, but please don't share the actual message or file further. New audio messages will be sent on Mondays (every week or every 2 weeks, depending on the study group you are assigned to), and you will receive Le10.000 data top up every time before receiving the audio message. It is important that your mobile phone and your WhatsApp are close to you and work properly in the next two months. Between December 2-14, we will visit you again, with some more health-related questions we would like to ask you. We will send you a message on WhatsApp to notify when we come. When you have completed the last survey, you will receive the last 10.000Le data top up.

It is important that you save the following number on your phone under the name 'Information Na Pawa': 030070104

This is the phone number that you will receive the audio messages from. You need to have this phone number in your address book on your phone, otherwise you will not be able to receive the audio messages.

Please do NOT answer to the messages you receive, we will not respond to messages written on WhatsApp. If you have questions or problems receiving the audio messages, please contact the study team on this phone number: 030070138

4. **Possible risks**

Because the study only involves answering questions and listening to audio messages, there is no personal risk for you to participate in this study.

5. **If you want to stop participating in the study**

Your participation is entirely voluntary, it is your choice if you want to participate or not. You can withdraw your participation at any moment in the study and you don't need to give a reason for doing so. There are no consequences if you decide to withdraw your consent. You can refuse to answer any questions.

6. **Usage and storage of your data**

Your personal data will be collected, analysed and stored for this study. This is data such as your name, address, phone number and educational level. We need to collect this personal data to be able to carry out this study. We ask your permission to use your personal data for this purpose. Karolinska Institutet is a public authority and is thereby obliged to comply with, among other things, the rules on public documents, public authority archives and public statistics. Karolinska Institutet will therefore also process personal data in the manner required to comply with other applicable legislation.

Confidentiality of your data

To protect your privacy, we will remove your name and other information that can be traced back to you when we analyse the data. Instead, we will give your data will a unique code, so that the data cannot be traced back to you. The encryption key remains safely stored at Karolinska Institutet and will only be accessible to authorized researchers who are directly involved in the analysis of the data for this study. The data cannot be traced back to you in reports and publications about the study.

Storage and use of data

Your data will be stored on safe servers at Karolinska Institutet in Sweden. Your personal data is handled in accordance with regulations regarding public authority archives. Collected personal data may be used in future research projects at Karolinska Institutet that have been approved by the Ethical Review Board. Your data will be stored for 10 years for reviewing purposes.

Withdrawing consent

You can withdraw your consent to the use of your personal data at any time. This applies to this study and also to storage and use for future research. The study data collected until the moment you withdraw your consent will still be used in the study.

7. **Compensation for participation**

If you decide to participate in this study, you will receive a data top up of Le10.000 on your phone every time you receive an audio message on WhatsApp. Depending on which study group you will be randomly assigned to, you will receive 2 or 4 audio messages. After completion of the follow-up survey, you will receive a final Le10.000. All participants who have completed the follow-up survey have an extra chance to win an additional Le30.000 phone top up.

8. **Any questions**

If you have any questions, please contact the study team: 030070138
Or email the study team members:
Maike Winters (Karolinska Institutet): maike.winters@ki.se
Helena Nordenstedt (Karolinska Institutet): helena.nordenstedt@ki.se
Paul Sengeh (FOCUS1000): psengeh@gmail.com

9. **Signing the consent form**

When you are sure you understand the above presented information, you will be asked to decide on participation in this study. If you give permission, we will ask you to confirm this in writing on the appended consent form. By your written permission, you indicate that you have understood the information and consent to participation in the study. Both yourself and the investigator will receive a signed copy of the consent form.

Consent Form Information Na Pawa Study

I have read the participant information form. I was able to ask questions. My questions have been answered to my satisfaction. I had enough time to decide whether to participate.
I know that participation is voluntary. I know that I may decide at any time not to participate after all or to withdraw from the study. I do not need to give a reason for this. I give permission for the collection and use of my data to answer the research question in this study.

I want to participate in this study.

Name of the participant:

Signature: Date:

……………………………………………………….

I hereby declare that I have fully informed the participant about this study. If information comes to light during the course of the study that could affect the participant's consent, I will inform him/her of this in a timely fashion.

Name of investigator / enumerator:

Signature: Date:

……………………………………………………….

I:
☐ do
☐ do not
consent to keeping my personal data longer and for it to be used for future research

Winters M, *et al. BMJ Global Health* 2021; 6:e006954. doi: 10.1136/bmjgh-2021-006954

Appendix C

Baseline Survey Contagious Misinformation Trial

1. Name of enumerator

2. Does the participant have WhatsApp?
    a. Yes
    b. No

3. Does the particpant want to be part of the study?
    a. Yes
    b. No -> end of survey

4. Is the study phone number programmed in the participant's phone?
    a. Yes → Q6
    b. No → Q5

5. If no, why not?
    a. Open

6. Did the participant sign the informed consent forms?
    a. Yes
    b. No -> end of survey

7. First Name:……….

8. Last Name:……..

9. Address

10. Describe house / address / landmark:

11. WhatsApp phone number:

12. Is your WhatsApp number from Orange, Africell or Qcell?
    a. Orange
    b. Africell
    c. Q cell
    d. Other:
    e. I don't know
    f. No answer

13. Other phone number participant can be reached on:

14. Gender:
    a. Male
    b. Female

15. Age: ………… years

16. What is your highest level of education?
    a. No formal education
    b. Primary education
    c. Secondary education
    d. Post-secondary education
    e. I don't know / not sure

f. No response

17. Average monthly household income from paid job: Le………………….

18. What is your religion?
    a. Islam
    b. Christianity
    c. Other: …………
    d. No response

Awareness / risk perception:

19. How often do you discuss health issues with family or friends?
    a. Daily
    b. Weekly
    c. Monthly
    d. When necessary
    e. Never
    f. I don't know / not sure
    g. No response

20. Do you know of any diseases that can be spread by being in contact with another person? Do not read the alternatives, select all that apply
    a. Malaria
    b. HIV
    c. Ebola
    d. Typhoid
    e. Measles
    f. Lassa Fever
    g. Cholera
    h. Zika
    i. Tuberculosis
    j. Leprosy
    k. Scabies
    l. Sexually transmitted diseases
    m. Other

21. Have you ever had typhoid?
    a. Yes: go to Q22
    b. No: go to Q25
    c. I don't know / not sure

22. How did you know you had typhoid? Do not read the alternatives, select all that apply
    a. Diagnosed in a hospital
    b. Diagnosed in a health facility other than hospital
    c. Diagnosed in a private clinic
    d. From a pharmacy
    e. From a health center
    f. From a non-health worker
    g. From a lab
    h. Somebody told me
    i. From the symptoms
    j. I don't know / not sure
    k. No response

23. How many times have you had typhoid?
    a.

24. When was the last time you had typhoid?
    a. In the last 3 months
    b. In the last 6 months
    c. In the last year
    d. Longer ago
    e. I can't remember
    f. No response

25. <u>For data collectors only:</u> Did the respondent mention 'typhoid-malaria' so far?
    a. Yes
    b. No

26. How does a person get typhoid? <u>Do not read the alternatives, select all that apply</u>
    a. Mosquitoes
    b. After getting malaria
    c. Bacteria
    d. Virus
    e. Parasite
    f. Witchcraft / Evildoing / Sin
    g. God or higher power
    h. By eating contaminated food
    i. By drinking contaminated water
    j. Eating with dirty hands
    k. Contact between houseflies and food
    l. Contact with vomit or stool
    m. Drinking too much beer
    n. Eating too many oranges
    o. Eating peanuts
    p. Eating oily foods
    q. Not washing off sweat
    r. Other:………………..
    s. I don't know / not sure
    t. No Response

27. Can a person get typhoid from mosquitoes?
    a. Yes
    b. No
    c. I don't know / not sure
    d. No response

28. How would you know that someone is infected with typhoid (signs and symptoms)? <u>Do not read the alternative, select all that apply</u>
    a. Weakness
    b. Fever
    c. Chills
    d. Headache
    e. Muscle pain
    f. Diarrhea
    g. Vomiting
    h. Abdominal (stomach) pain
    i. Constipation
    j. Lack of appetite
    k. Sore throat
    l. Cough
    m. Rash
    n. Difficulty breathing

o. Confusion
p. Dizziness
q. Yellow eyes / yellow urine
r. Other:….
s. I don't know / not sure
t. No response

29. Can you name up to three ways how you can prevent yourself from getting typhoid? Do not read the alternative, select all that apply (max three!)
    a. Sleep under a bednet
    b. Drink treated water
    c. Wash hands with soap before eating
    d. Wash hands before cooking food
    e. Wash hands after leaving the toilet
    f. Wash food before eating
    g. Cook foods thorougly
    h. Eat foods while hot
    i. Take anti-malarials
    j. Don't eat too many oranges
    k. Don't eat oily food
    l. Don't drink too much beer
    m. Keep environment clean
    n. Other:
    o. I don't know / not sure
    p. No response

30. If you think that you or someone in your family has typhoid, what would you do? Do not read the alternative, select all that apply
    a. Go to a health facility / hospital
    b. Go to a traditional healer, or traditional birth attendant
    c. Get medication from a pharmacy
    d. Get antibiotics from a pharmacy
    e. Get antimalarials from a pharmacy
    f. Self-medicate
    g. Other:
    h. I don't know / not sure
    i. No Response

31. Let's say you keep doing what you already do to avoid typhoid, do you think it is likely or unlikely for you to get typhoid in the next year?
    a. Likely
    b. Unlikely
    c. I don't know
    d. No response

32. Do you currently take actions to avoid getting typhoid?
    a. Yes
    b. No → Q34
    c. I don't know
    d. No response

33. What kind of actions do you currently take to avoid getting typhoid? Do not read the alternatives, select all that apply
    a. Sleep under a bednet
    b. Drink treated water
    c. Wash hands with soap before eating
    d. Wash hands before cooking food

e. Wash hands after leaving the toilet
f. Wash food before eating
g. Cook foods thorougly
h. Eat foods while hot
i. Take anti-malarials
j. Don't eat too many oranges
k. Don't eat oily food
l. Don't drink too much beer
m. Keep environment clean
n. Other:
o. I don't know / not sure
p. No response

34. Would you take actions in the next year to avoid getting typhoid?
    a. Yes
    b. No → Q36
    c. I don't know
    d. No response

35. What kind of actions would you take in the next year to avoid getting typhoid? <u>Do not read the alternatives, select all that apply</u>
    a. Sleep under a bednet
    b. Drink treated water
    c. Wash hands with soap before eating
    d. Wash hands before cooking food
    e. Wash hands after leaving the toilet
    f. Wash food before eating
    g. Cook foods thorougly
    h. Eat foods while hot
    i. Take anti-malarials
    j. Don't eat too many oranges
    k. Don't eat oily food
    l. Don't drink too much beer
    m. Keep environment clean
    n. Other:
    o. I don't know / not sure
    p. No response

36. How does a person get malaria? <u>Do not read the alternatives, select all that apply</u>
    a. Mosquitoes
    b. Bacteria
    c. Virus
    d. Parasite
    e. Witchcraft / Evildoing / Sin
    f. God or higher power
    g. By eating contaminated food
    h. By drinking contaminated water
    i. Eating with dirty hands
    j. Contact between houseflies and food
    k. Contact with vomit or stool
    l. Drinking too much beer
    m. Eating too many oranges
    n. Eating Peanuts
    o. Eating Oily foods
    p. Not washing off sweat
    q. Other:………………..
    r. I don't know / not sure

s. No Response

37. Can you get typhoid without getting malaria?
    a. Yes; Q40
    b. No; Q38
    c. I don't know
    d. No response

38. Why do you think that? Do not read the alternative, select all that apply
    a. Typhoid and malaria go together
    b. First you get malaria, then typhoid
    c. Typhoid is a bad form of malaria
    d. Both are caused by mosquitoes
    e. Signs and symptoms are the same
    f. Other:

39. Where did you get this information? Do not read the alternatives, select all that apply
    a. Health facility / Hospital
    b. Radio
    c. Television
    d. Church / mosque / other religious venues
    e. Community meetings
    f. Newspapers / brochures / other print material
    g. Mobile phones / text messages
    h. Traditional leaders
    i. Traditional healers
    j. Government / Ministry of Health / Well Bodi Ministry
    k. Family/friends
    l. Doctors
    m. Nurses
    n. Other health workers
    o. Other community workers
    p. Other:
    q. I don't know / not sure
    r. No Response

40. Have you heard of typhoid-malaria?
    a. Yes
    b. No
    c. I don't know / not sure
    d. No response

41. Geo location

Follow-up survey Contagious Misinformation Trial
Version 2, September 2019

1. First Name:……..

2. Last Name:………

3. WhatsApp phone number

Awareness / risk perception

4. How often do you discuss health issues with family or friends? Do not read the alternatives, select all that apply
    a. Daily
    b. Weekly
    c. Monthly
    d. When necessary
    e. Never
    f. I don't know / not sure
    g. No response

5. Do you know of any diseases that can be spread by being in contact with another person?
    a. Malaria
    b. HIV
    c. Ebola
    d. Typhoid
    e. Measles
    f. Lassa Fever
    g. Cholera
    h. Zika
    i. Tuberculosis
    j. Leprosy
    k. Scabies
    l. Sexually transmitted diseases
    m. Other

6. Have you had typhoid in the last 2 months?
    a. Yes: go to Q7
    b. No: go to Q9
    c. I don't know / not sure

7. How did you know you had typhoid? Do not read the alternatives, select all that apply
    a. Diagnosed in a hospital
    b. Diagnosed in a health facility other than hospital
    c. Diagnosed in a private clinic
    d. From a pharmacy
    e. From a health center
    f. From a non-health worker
    g. From a lab
    h. Somebody told me
    i. From the symptoms

   j. I don't know / not sure
   k. No response

8. Do you think it is likely or unlikely for you to get typhoid in the next year?
   a. Likely
   b. Unlikely
   c. I don't know
   d. No response

9. For data collectors only: Did the respondent mention 'typhoid-malaria' so far?
   a. Yes
   b. No

Knowledge, attitudes, practices:

10. Have you taken actions to avoid getting typhoid?
    a. Yes
    b. No: Q12
    c. I don't know
    d. No response

11. In what ways have you taken actions to avoid getting typhoid? Do not read the alternatives, select all that apply
    a. Sleep under a bednet
    b. Drink treated water
    c. Wash hands with soap before eating
    d. Wash hands before cooking food
    e. Wash hands after leaving the toilet
    f. Wash food before eating
    g. Cook foods thorougly
    h. Eat foods while hot
    i. Take anti-malarials
    j. Don't eat too many oranges
    k. Don't eat oily food
    l. Don't drink too much beer
    m. Clean environment
    n. Other:
    o. I don't know / not sure
    p. No response

12. Would you take actions in the next year to avoid getting typhoid?
    a. Yes
    b. No: Q 14
    c. I don't know
    d. No response

13. What kind of actions would you take in the next year to avoid getting typhoid? Do not read the alternatives, select all that apply
    a. Sleep under a bednet
    b. Drink treated water
    c. Wash hands with soap before eating
    d. Wash hands before cooking food

e. Wash hands after leaving the toilet
f. Wash food before eating
g. Cook foods thorougly
h. Eat foods while hot
i. Take anti-malarials
j. Don't eat too many oranges
k. Don't eat oily food
l. Don't drink too much beer
m. Clean environment
n. Other:
o. I don't know / not sure
p. No response

14. How does a person get typhoid? <u>Do not read the alternatives, select all that apply</u>
    a. Mosquitoes
    b. After getting malaria
    c. Bacteria
    d. Virus
    e. Parasite
    f. Witchcraft / Evildoing / Sin
    g. God or higher power
    h. By eating contaminated food
    i. By drinking contaminated water
    j. Eating with dirty hands
    k. Contact between houseflies and food
    l. Contact with vomit or stool
    m. Drinking too much beer
    n. Eating too many oranges
    o. Eating peanuts
    p. Eating oily foods
    q. Other:……………….
    r. I don't know / not sure
    s. No Response

15. Can a person get typhoid from mosquitoes?
    a. Yes
    b. No
    c. I don't know / not sure
    d. No response

16. How would you know that someone is infected with typhoid (signs and symptoms)? Do <u>not read the alternative, select all that apply</u>
    a. Weakness
    b. Fever
    c. Chills
    d. Headache
    e. Muscle pain
    f. Diarrhea
    g. Vomiting
    h. Abdominal (stomach) pain
    i. Constipation
    j. Lack of appetite

k. Sore throat
l. Cough
m. Rash
n. Difficulty breathing
o. Confusion
p. Dizziness
q. Yellow eyes / yellow urine
r. Other:….
s. I don't know / not sure
t. No response

17. Can you name up to three ways how you can prevent yourself from getting typhoid? Do not read the alternative, select all that apply
    a. Sleep under a bednet
    b. Drink treated water
    c. Wash hands with soap before eating
    d. Wash hands before cooking food
    e. Wash hands after leaving the toilet
    f. Wash food before eating
    g. Cook foods thorougly
    h. Eat foods while hot
    i. Take anti-malarials
    j. Don't eat too many oranges
    k. Don't eat oily food
    l. Don't drink too much beer
    m. Clean environment
    n. Other:
    o. I don't know / not sure
    p. No response

18. If you think that you or someone in your family has typhoid, what would you do? Do not read the alternative, select all that apply
    a. Go to a health facility / hospital
    b. Go to a traditional healer, or traditional birth attendant
    c. Get medication from a pharmacy
    d. Get antibiotics from a pharmacy
    e. Get antimalarials from a pharmacy
    f. Self-medicate
    g. Other:
    h. I don't know / not sure
    i. No Response

19. How does a person get malaria? Do not read the alternatives, select all that apply
    a. Mosquitoes
    b. Bacteria
    c. Virus
    d. Parasite
    e. Witchcraft / Evildoing / Sin
    f. God or higher power
    g. By eating contaminated food
    h. By drinking contaminated water
    i. Eating with dirty hands

j. Contact between houseflies and food
k. Contact with vomit or stool
l. Drinking too much beer
m. Eating too many oranges
n. Eating Peanuts
o. Eating Oily foods
p. Not washing off sweat
q. Yellow eyes / yellow urine
r. Other:……………….
s. I don't know / not sure
t. No Response

20. Can you get typhoid without getting malaria?
    a. Yes; Q28
    b. No; Q26
    c. I don't know
    d. No response

21. Why do you think that? <u>Do not read the alternative, select all that apply</u>
    a. Typhoid and malaria go together
    b. First you get malaria, then typhoid
    c. Typhoid is a bad form of malaria
    d. Both are caused by mosquitoes
    e. Signs and symptoms are the same
    f. Other:

22. Where did you get this information? <u>Do not read the alternatives, select all that apply</u>
    a. Health facility / Hospital
    b. Radio
    c. Television
    d. Church / mosque / other religious venues
    e. Community meetings
    f. Newspapers / brochures / other print material
    g. Mobile phones / text messages
    h. Traditional leaders
    i. Traditional healers
    j. Government / Ministry of Health / Well Bodi Ministry
    k. Family/friends
    l. Doctors
    m. Nurses
    n. Other health workers
    o. Other community workers
    p. Other:
    q. I don't know / not sure
    r. No Response

23. Have you heard of typhoid-malaria?
    a. Yes
    b. No
    c. I don't know / not sure
    d. No response

Audio messages:

24. How many audio messages did you get? Was it 2 or 4 audio messages?

25. How many audio messages did you listen to?

26. Whas the audio about typhoid or about breastfeeding?
    a. About typhoid (go to Q37)
    b. About breastfeeding (go to Q40)

27. Who was the main character in the drama?
    a. Mariama
    b. Abu
    c. Sullay
    d. Other:

28. What happened to the main character in the drama?
    a. Is the participant describing it correctly?
        i. Yes
        ii. No
        iii. Not sure
    b. If not sure: describe what participant says

29. Did you discuss the audio fragments with friends and/or family?
    a. Yes
    b. No

30. Did you like the drama?
    a. Yes
    b. No

31. Have you talked to anyone who has heard or received these messages as well?
    a. Yes
    b. No
    c. Other:

32. Geo location
